# Melanin‐based color variation in response to changing climates in snakes

**DOI:** 10.1002/ece3.11627

**Published:** 2024-06-28

**Authors:** J. Goldenberg, K. Bisschop, G. Bruni, M. R. Di Nicola, F. Banfi, F. P. Faraone

**Affiliations:** ^1^ Division of Biodiversity and Evolution, Department of Biology Lund University Lund Sweden; ^2^ Evolution and Optics of Nanostructures Group, Department of Biology Ghent University Ghent Belgium; ^3^ Laboratory of Aquatic Biology KU Leuven Kulak Kortrijk Belgium; ^4^ Terrestrial Ecology Unit, Department of Biology Ghent University Ghent Belgium; ^5^ Independent Researcher, Viale Palmiro Togliatti Sesto Fiorentino Florence Italy; ^6^ Faculty of Veterinary Medicine, Department of Pathobiology, Pharmacology and Zoological Medicine, Wildlife Health Ghent Ghent University Merelbeke Belgium; ^7^ Unit of Dermatology and Cosmetology IRCCS San Raffaele Hospital Milan Italy; ^8^ Laboratory of Functional Morphology, Department of Biology University of Antwerp Wilrijk Belgium; ^9^ Dipartimento Scienze e Tecnologie Biologiche, Chimiche e Farmaceutiche University of Palermo Palermo Italy

**Keywords:** intraspecific variation, melanism, micro‐to‐macro evolution, time series

## Abstract

Melanism, the process of heavier melanin deposition, can interact with climate variation at both micro and macro scales, ultimately influencing color evolution in organisms. While the ecological processes regulating melanin production in relation to climate have been extensively studied, intraspecific variations of melanism are seldom considered. Such scientific gap hampers our understanding of how species adapt to rapidly changing climates. For example, dark coloration may lead to higher heat absorption and be advantageous in cool climates, but also in hot environments as a UV or antimicrobial protection mechanism. To disentangle such opposing predictions, here we examined the effect of climate on shaping melanism variation in 150 barred grass snakes (*Natrix helvetica*) and 383 green whip snakes (*Hierophis viridiflavus*) across Italy. By utilizing melanistic morphs (charcoal and picturata in *N. helvetica*, charcoal and abundistic in *H. viridiflavus*) and compiling observations from 2002 to 2021, we predicted that charcoal morphs in *H. viridiflavus* would optimize heat absorption in cold environments, while offering protection from excessive UV radiation in *N. helvetica* within warm habitats; whereas picturata and abundistic morphs would thrive in humid environments, which naturally have a denser vegetation and wetter substrates producing darker ambient light, thus providing concealment advantages. While picturata and abundistic morphs did not align with our initial humidity expectations, the charcoal morph in *N. helvetica* is associated with UV environments, suggesting protection mechanisms against damaging solar radiation. *H. viridiflavus* is associated with high precipitations, which might offer antimicrobial protection. Overall, our results provide insights into the correlations between melanin‐based color morphs and climate variables in snake populations. While suggestive of potential adaptive responses, future research should delve deeper into the underlying mechanisms regulating this relationship.

## Graphical abstract

Melanism, characterized by increased melanin deposition, interacts with climate variation, influencing color evolution in organisms. Despite extensive study of ecological processes regulating melanism production, intraspecific variations are often overlooked, impeding our understanding of species' responses to changing climates. Examining 150 barred grass snakes and 383 green whip snakes across Italy, we found that charcoal morphs are selected in cold environments likely for heat absorption and in warm habitats possibly offering protection from UV radiation, while picturata and abundistic morphs thrive in humid environments, suggesting complex relationships between melanism and climate variables in snake populations.

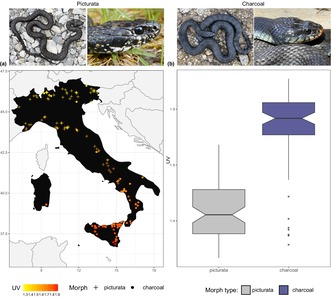

## 1 INTRODUCTION

Intraspecific variation, often described as the inherent variation within a single species (Harding et al., 2019), is essential for deciphering the driving forces behind local adaptation processes because it provides a window into the selective pressures acting on different populations (Mader et al., 2022). Variations in traits such as morphology, physiology, behavior, and genetic makeup may emerge as populations respond to different ecological conditions, such as fluctuations in temperature (Jonsson et al., 2022), precipitation (McCain, 2009), food availability (Kumar et al., 2022), and predation pressures (Vervust et al., 2007). Moreover, intraspecific variation contributes significantly to the overarching framework of biodiversity, promoting ecological stability, driving speciation and extinction processes, and leading to the rise of ecotypes (or morphs) (Mimura et al., 2017). By studying these variations, it is possible to underpin the specific genetic and phenotypic changes that occur in response to environmental changes. This helps to clarify the roles of different selective pressures and how they shape the adaptive responses of local populations. Furthermore, understanding these interactions allows to predict the resilience of populations to future environmental changes and provides insights into their evolutionary trajectories and success (Barabás & D'Andrea, 2016).

Among the drivers of intraspecific variation, environmental factors are arguably the most prominent because they may influence the foundational dynamics of an ecosystem web (Whitham et al., 2006), impacting the viability of populations and shaping their genetic diversity (Lande, 1988). Abiotic factors such as temperature, precipitation, humidity, wind speed, and elevation gradient have all been identified as motors of local adaptation (Hereford, 2009), shaping the physiology, behavior, reproduction, and dispersal of organisms by influencing traits such as body size (Yom‐Tov & Geffen, 2011), coloration (Clusella‐Trullas et al., 2008) and metabolic rates (Cano & Nicieza, 2006). Although these traits may interact to shape local adaptation (Buckley et al., 2014), within a single ecosystem, microhabitats may vary in terms of color and light conditions, ultimately inducing selection of specific color patterns to enhance camouflage (McLean et al., 2014), protect against harmful UV radiation (Rogalski & Duffy, 2020), or for thermoregulation (Refsnider et al., 2018).

One way to study how micro‐and‐macro processes are linked together is through time series analyses, which provide temporal windows to track how focal traits respond to ecological and evolutionary pressures (Hendry & Kinnison, 1998). Although such analyses focus on limited timescales compared to geological periods, they can provide valuable insights into short term adaptation processes. For example, introduced lizards onto small islands have been shown to diverge morphologically from the nearby source as an adaptation response to changing habitats in only 10–14 years (Losos et al., 1997). Even over shorter time scales, extreme weather events like hurricanes have been demonstrated to serve as selective agents, favoring the inheritance of life‐saving traits when exposed to these conditions (Donihue et al., 2020). By understanding the drivers behind such evolutionary adaptations, it is possible to evaluate how plastic traits are under different temporal scales. Including such evolutionary dimension can improve species resilience modeling by accounting for key processes such as adaptive responses to changing environmental conditions (Waldvogel et al., 2020). Recent research has indicated that by the end of this century, variations in melanin levels among different populations of lizards are expected to have differential effects on their activity patterns, due to projected temperature and precipitation fluctuations at the microclimate level (Mader et al., 2022). These microevolutionary processes are integral to shaping the macroevolution of species, favoring adaptive coloration and promoting intraspecific diversity at the local scale.

Melanin, which encompasses both eumelanin and pheomelanin, is a ubiquitous pigment with multifaceted functions (Xie et al., 2024), subject to profound ecological significance (Goldenberg et al., 2022). While our study primarily focuses on eumelanin, which we refer to as “melanin,” it is important to recognize the broader context of melanin diversity. While eumelanin is associated with darker pigmentation and tends to increase in concentration in response to higher levels of UV radiation and sunlight exposure, pheomelanin is linked to responses to oxidative stress and may be more prevalent in environments with lower UV radiation levels (Nasti & Timares, 2015). Climatic factors' influence on melanin levels is extensively studied through ecogeographical hypotheses. Among them, only the thermal‐melanism hypothesis (Bogert, 1949; Clusella‐Trullas et al., 2007) and Gloger's rule (Gloger, 1833; Rensch, 1929), elucidates pigmentation variation across geographic gradients. Gloger's rule posits that animals in warmer and wetter climates will exhibit darker pigmentation (Gloger, 1833; Rensch, 1929), which provides protection against parasites, UV radiation, or aids in camouflage (Delhey, 2019). On the other hand, the thermal‐melanism hypothesis (Bogert's rule) proposes that animals in colder regions will have darker pigmentation to aid in thermoregulation (Bogert, 1949; Clusella‐Trullas et al., 2007). For example, UV radiation, particularly in high doses, poses a potential threat to an organism's genetic material and cellular integrity (Roy, 2017). In response, melanin often acts as a natural sunscreen, shielding against the harmful effects of UV radiation (Wakamatsu & Ito, 2021). Consequently, melanin levels can vary in response to UV intensity, with organisms inhabiting sunnier regions frequently displaying melanism (i.e. heavier deposition of melanin due to increased melanogenesis) as an adaptive response (Nicolaï et al., 2020). Conversely, in cold environments, dark melanin coloration may provide thermal benefits by assisting in heat absorption (Clusella‐Trullas et al., 2007; Martínez‐Freiría et al., 2020). Elevation can further compound these effects (Reguera et al., 2014), as higher altitudes often coincide with lower temperatures and increased UV radiation exposure. Precipitation and humidity patterns are another critical factor influencing melanin production. Higher precipitation levels, especially in warm environments, may promote bacterial infections, and melanism confer antimicrobial protection (Mackintosh, 2001), whereby melanin can act as a protection shield to isolate external pathogens such as fungal spores (Mackintosh, 2001). In addition, melanin plays a crucial role in camouflage mechanisms, where heavily pigmented melanin‐based coloration helps organisms blend into their surroundings, especially in dark, humid habitats (Delhey, 2019), providing effective means of concealment from predators or prey. Although less frequently studied, wind speed can affect melanin levels in organisms living in exposed habitats. For example, strong winds have been shown to contribute to desiccation and heat loss in ectotherms (Ortega et al., 2017), which may influence the selective pressures favoring melanism in windy environments (Jong et al., 1996). Intriguingly, the interplay between these climate variables and melanism is highly context‐dependent and can lead to divergent adaptive strategies across and within species (Roulin, 2014). Ectotherms may be particularly vulnerable to local environmental factors because they depend on the surrounding environment for thermoregulation (Clusella‐Trullas et al., 2007). As such, melanism is likely to exert a stronger influence in these organisms than in endotherms (Goldenberg et al., 2022), which primarily rely on metabolic processes to regulate their body temperature.

**FIGURE 1:**
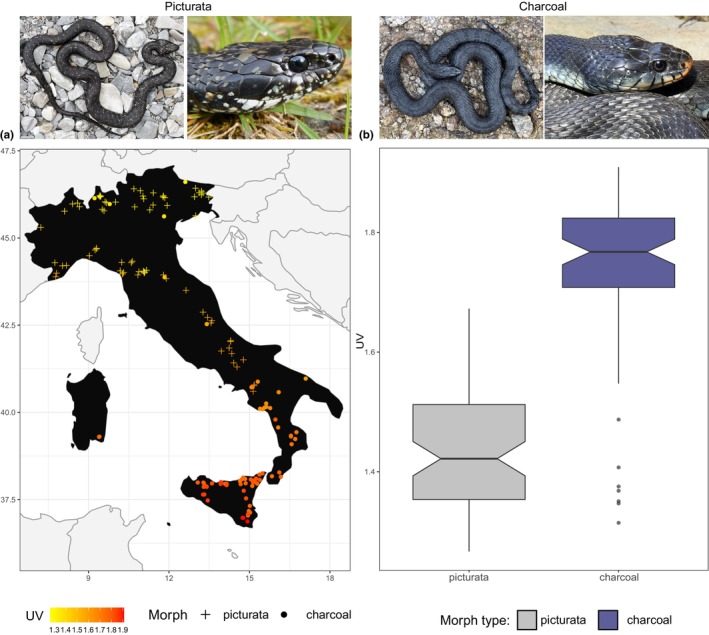
Relationship between UV radiation and melanin‐rich morph types in *N. helvetica*. (a) Across the Italian peninsula, charcoal morphs cluster in the southern regions with stronger UV radiations, whereas picturata with lower UV radiations. (b) The box plot with notches shows that charcoal morphs overlap with picturata, and not vice versa, but picturata is selected in lower UV radiation regions. Specimen photos adapted with permission from Bruni et al. (2022).

**FIGURE 2:**
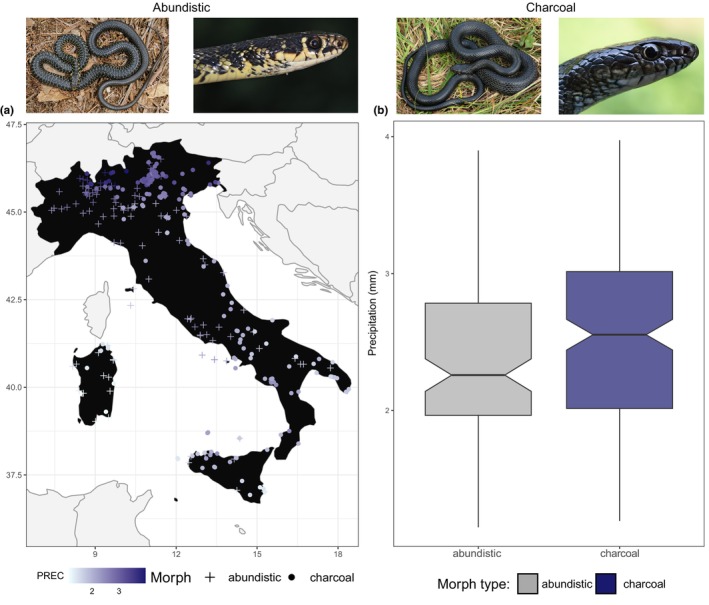
Relationship between precipitation patterns and melanin‐rich morph types in *H. viridiflavus*. (a) Across the Italian peninsula, charcoal morphs cluster in the southern regions and north‐eastern ward, whereas abundistic in central islands and north‐western ward. (b) The box plot with notches shows that charcoal morphs overlap with abundistic, and to a lesser extent vice versa, but abundistic is favored in lower precipitation pattern areas (notches not overlapping). Specimen photos from MRDN.

Snakes are one such group for which the ecology and evolutionary dynamic of the colored integument in relation to climate have undergone extensive scrutiny. However, intraspecific variations of melanism are less often considered (but see e.g. Mader et al., 2022), which could provide valuable insights into the adaptation mechanisms of populations to changing environments (Roulin, 2014). While some studies support the notion that melanin‐based coloration has evolved in response to climate change (e.g. MacLean et al., 2019), further data are needed to understand the underlying processes behind such evolutionary trend (Clusella‐Trullas & Nielsen, 2020). Here, we delved into the influence of climate on shaping melanism variation in two snake species, the barred grass snakes *Natrix helvetica* (Lacépède, 1789) and the green whip snakes *Hierophis viridiflavus* (Lacépède, 1789), inhabiting various regions across the Italian peninsula. We focused on their melanistic morphs, the “charcoal” (characterized by a uniform dark coloration) and the “picturata” (featuring a speckled black pattern) morphs in *N. helvetica* (Figure 1; see Bruni et al., 2022) and the “charcoal” and “abundistic” (showing a widening in the dark elements of the dorsal pattern such as blotches, spots and stripes; see Zuffi, 2008) morphs in *H. viridiflavus* (Figure 2). Our investigation drew upon observational data spanning nearly two decades, from 2002 to 2021. Overall, we aimed to provide insights into the ecogeographic distribution of different melanistic morph types in response to changing climates, considering the complexities associated with intraspecific melanism variations. Given their geographic distribution (Figures 1a and 2a), we specifically posited that charcoal morphs might be favored in cold environments for enhanced heat absorption, particularly in *H. viridiflavus*, while simultaneously providing protection against excessive UV radiation in warm habitats, particularly in *N. helvetica*. Conversely, we predicted that picturata and abundistic morphs would thrive in humid environments, benefiting from their dark, speckled, or abundistic coloration, which not only aids in camouflage within such densely vegetated regions but also serves as a shield mechanism against pathogens. This expectation stems from the idea that a dark disruptive coloration is more suited for concealment in complex environments than a plain dark color (Adams et al., 2019). Therefore, we believe that picturata and abundistic morphs, with their relatively dark and melanin‐rich coloration, likely have dual benefits: antimicrobial protection and effective concealment. These insights align with established ecogeographical principles such as Bogert (Clusella‐Trullas et al., 2007) and Gloger's (Delhey, 2019) rules, offering valuable perspectives on the interplay between environmental factors and melanin expression across diverse populations.

## 2 MATERIALS AND METHODS

### 2.1 Species selection and dataset construction

To elucidate how climatic variables are associated with the geographical distribution of melanin‐rich morphs, we selected two aglyphous, diurnal snakes from different colubrid families (Di Nicola et al., 2021; Zaher et al., 2019). The barred grass snake (*N. helvetica*), with its diverse morphological variations (Kindler et al., 2017), wide distribution across the Italian peninsula and a relative long evolutionary path (7 million years—My—since its split with the extant sister species *N. astreptophora*; Schöneberg et al., 2023), is an ideal study organism for investigating the influence of local climate on melanin levels. The charcoal morph is predominantly distributed in southern Italy, Sicily, and Sardinia, whereas picturata in the central‐northern part of the peninsula (Bruni et al., 2022; Di Nicola, Chiara, et al., 2023). Similarly, the green whip snake (*H. viridiflavus*) is widely distributed across Italy (Storniolo et al., 2023), presents two clades currently recognized as subspecies (Di Nicola et al., 2021; Sindaco & Razzetti, 2021), and also has a long evolutionary path (8 My since its split with the extant sister species *H. gemonensis*; Kumar et al., 2017). Importantly, color morphs and subspecies are not synonymous, as morph distinctions are primarily delineated based on geographical distributions (Storniolo et al., 2023). While both species exhibit structural similarities in their morphology, such as similar body shapes and sizes, they demonstrate distinct behavioral differences. *N. helvetica* often displays defensive behaviors such as musking, characterized by the release of biproducts from their digestive system via the cloaca, feigning death (known as thanatosis), and very rarely bites (Di Nicola, Pozzi, et al., 2023; Gregory et al., 2007), whereas *H. viridiflavus* tends to exhibit more aggressive behaviors when threatened (Di Nicola et al., 2021; Dutto et al., 2015). These behavioral differences may reflect variations in their ecological niches and predator–prey interactions.

For *N. helvetica*, we collected observation data on 150 individuals (77 picturata, 73 charcoal) from our long‐term dataset, which includes a combination of authors and citizen science records (see Bruni et al., 2022) spanning from 2002 to 2021. For each location we extracted (a) observation date; (b) latitude and longitude; (c) altitude; (d) morph type; and compiled (e) microclimate variables known to influence the colored integument of other ectothermic species (i.e. UV, temperature, wind speed, precipitation, and humidity; Kearney & Porter, 2009). Despite the elusive nature of snakes, leading to sampling underestimations, the database was meticulously compiled from various sources, including opportunistic surveys by the authors, inquiries with collaborators, consultation with field zoologists from different Italian provinces, and an extensive review of the literature. In addition, we conducted a thorough screening of records on major citizen science platforms (i.e. iNaturalist.org and Ornitho.org) and social networks such as Facebook, particularly within the group “Identificazione anfibi e rettili” (https://www.facebook.com/groups/283231695476830) and “Fauna Siciliana” (https://www.facebook.com/groups/283231695476830). This comprehensive approach aimed to bridge the gap between our dataset and the actual presence of melanistic individuals across the entire Italian territory. For *H. viridiflavus* we compiled the same data, but we sourced the observations only from iNaturalist (accessed in October 2023), filtering for coordinates with precisions of <1 km, on 316 individuals (198 charcoal, 118 abundistic) from 2006 to 2021 (prior 2006 observations are limited). To avoid repeated observations, we further refined our search by crosschecking different criteria such as location, date, and observer.

As *N. helvetica* and *H. viridiflavus* exhibit a lifespan of up to 20 years (AnAge: The Animal Aging and Longevity Database; accessed on February 24, 2023; Fornasiero et al., 2016), for each observation, we sourced microclimate data from the NASA Langley Research Centre (LaRC) POWER Project (https://power.larc.nasa.gov/data‐access‐viewer/) using the MERRA‐2 and CERES databases for 10 years prior to the observation date to represent average environmental conditions encountered by individuals within their geographic range. We compiled climate averages for the months of April–September, which correspond to the most active periods in the life cycle of these species. Such an approach allowed us to assemble a tailored, species‐specific microclimate dataset. Specifically, for each observation we collected UVA (W/m^2^), UVB (W/m^2^), UV (adimensional), mean ground temperature (°C) (temperature at the Earth's surface including the vegetated ground coverage), mean minimum temperature (*T*_min_,°C), mean maximum temperature (*T*_max_,°C), altitude (m), and precipitation (mm), along with wind speed (m/s) and humidity (%) at 2 m height.

### 2.2 Spatial and statistical analyses

To assess whether the selected environmental parameters influence the selection of morph type we first tested for spatial autocorrelation in the residuals—all analyses were performed in R v. 4.1.3 (R Core Team, 2023). As we found strong spatial patterns for each species, we performed generalized linear mixed models with “spaMM” (Rousset & Ferdy, 2014) using Maximum Likelihood and setting morph type as the dependent (outcome) variable, climate variables as independent (fixed) effects, observation year, and Matern effect (a correlation list between coordinates) as random factors. We selected binomial as family distribution, tested for comparisons between groups with the *ANOVA* function, and conducted model diagnostic in “DHARMa” (Hartig, 2022) (Figures S1 and S2). As UVA and UVB were highly autocorrelated (*R* = 0.98; *p* << .01), we ran all our models using UV, which includes both wavelengths. Similarly, humidity and precipitation (*R* = 0.80; *p* << .01), *T*_min_ and temperature (*R* = 0.85; *p* << .01), and *T*_max_ and temperature (*R* = 0.79; *p* << .01) were also highly autocorrelated. While humidity is an important factor related to moisture content in the air, it does not provide as direct or diverse ecological cues as precipitation, which encompasses the actual delivery of water to the landscape. Thus, we excluded humidity from the main analyses and included it in the supplementary materials for completeness. Furthermore, while extreme temperatures certainly influence phenotypic responses to climate change and are fundamental for biophysical models (Mader et al., 2022), average temperatures tend to be more stable and show clearer trends over time compared to the more variable minimum and maximum temperatures. This stability makes it easier to detect and analyze long‐term climatic shifts and their impacts, offering more comprehensive and ecologically relevant insights into how organisms and ecosystems respond to climatic shifts (Parmesan, 2006). Consequently, we presented the analyses using average temperature in the main text and included *T*_min_ and *T*_max_ in the supplementary materials to offer additional layers of understanding.

To examine how the relationship between climate variables and morph types changed over time, we subset our dataset in six sliding temporal windows of 10 years each (i.e. one year difference between windows). These subsets allowed us to capture average climate variations at finer temporal scales, while accounting for the average life span of *N. helvetica* and *H. viridiflavus*, and evaluate whether the significance of the examined variables changed across different periods. We utilized consistent spatial model structures as previously employed, thereby ensuring the consistency of dependent, independent, and random effects, as well as the binomial family distribution, across our analyses. For only these analyses, we considered observations starting from 2007 in *N. helvetica* and from 2006 in *H. viridiflavus*, because of a relatively low sample size for earlier periods hampering model convergence.

## 3 RESULTS

### 3.1 Spatial autocorrelations

**FIGURE 3:**
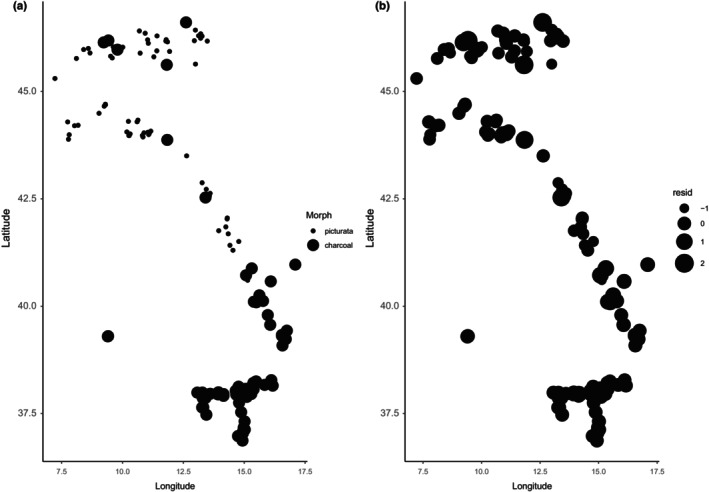
Spatial autocorrelation in *N. helvetica*. Spatial patterns in morph type (a) and the residuals (b) show regional clustering, warranting the use of spatial regression models to analyze the data.

**FIGURE 4:**
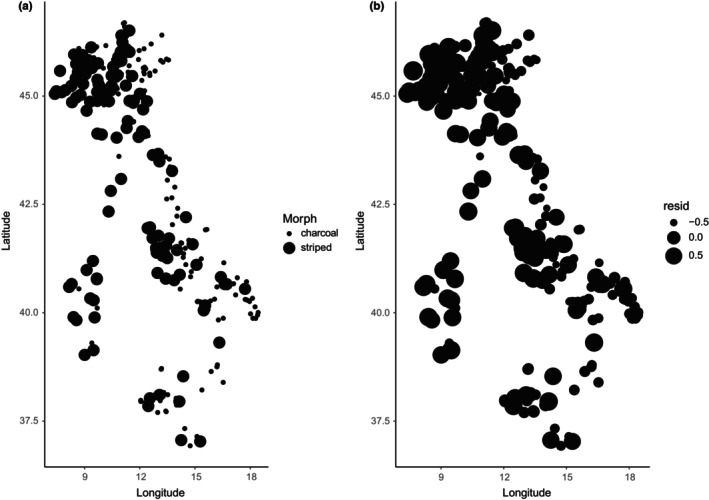
Spatial autocorrelation in *H. viridiflavus*. Spatial patterns in morph type (a) and the residuals (b) show regional clustering, warranting the use of spatial regression models to analyze the data.

As a first step, we tested for spatial autocorrelation in the residuals. We found strong spatial patterns (joint count in *N. helvetica*: picturata = 6.65, *p* << .01; charcoal = 4.04, *p* << .01; and in *H. viridiflavus*: picturata = 2.92, *p* < .01; abundistic: 2.03, *p* = .02; Moran's test for distance‐based autocorrelation in *N. helvetica* & *H. viridiflavus*: *p* << .01; Figures 3 and 4), warranting the use of spatial analyses.

### 3.2 Spatial associations

Then, we assessed which climate variables influence the selection of morph type. Of the five analyzed climate variables, only UV significantly influences morph type in *N. helvetica* (*χ* ^2^ = 5.9775; *p* < .01; Table S1), with high UV radiation selecting for the charcoal morph, whereas low UV radiation for picturata (Figures 1 and S3). We also found a significant effect of the intercept (*χ* ^2^ = 6.5623; *p* < .01), suggesting that other nonanalyzed variables drive the variation in the system. Differently from *N. helvetica*, precipitation patterns significantly influence morph type in *H. viridiflavus* (*χ* ^2^ = 5.38; *p* = .02; Table S2), with high‐precipitations favoring the charcoal morph and lows for abundistic (Figures 2 and S4). Interestingly, for both species, we retrieved a high smoothness parameter (*ν* = 16; Tables S1 and S2), indicating that the spatial process is particularly smooth and thus the correlation between points remains high over large distances, leading to very gradual changes in the process across space.

When evaluating the effects of *T*_min_ and *T*_max_ on morph types, we did not find any significant effect for *N. helvetica* (Tables S3–S6), suggesting that mean temperature provides a more integrated and relevant measure of the thermal environment affecting morph type. For *H. viridiflavus* we found that only precipitation significantly affects morph types but only when not accounting for interaction effects between *T*_min_ or *T*_max_ and precipitation (Tables S7–S10). We also conducted analyses using humidity instead of precipitation, and our results showed no significant effects except for a marginal interaction between average temperature and humidity in *H. viridiflavus* (Tables S11–S14).

To evaluate whether the divergence in outcomes between the two species is not attributed to differences in model structure, we produced two additional analyses, one for each species, accounting for the interaction between temperature and precipitation as a fixed effect. As we found diverging results between the two models (Table S15), the absence of temperature–precipitation interaction in our study does not explain for the disparities observed between species.

Since we also did not detect overall deviations from the expected distribution in the qq‐plot and that the model residuals against the predicted values are not deviate from expectations (Figures S1 and S2), our results are robust against perturbations in model structure, geographical, morph, and climate data distributions.

### 3.3 Phenotypic variations through time and space

**FIGURE 5:**
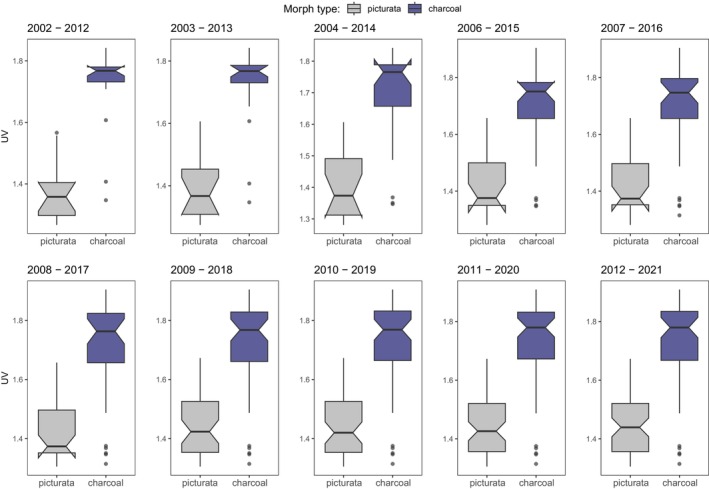
Morph type selection regulated by UV radiation in *N. helvetica*. Here, we show that the relationship between UV radiation and morph types does not change over time, where charcoal is persistently favored in high UV radiation regions, and vice versa for picturata. Note that for the statistical analyses we considered only periods following 2007 due to limited data hampering model convergence. Thus, box plots with notches prior to 2007 are for illustrative purposes only.

**FIGURE 6:**
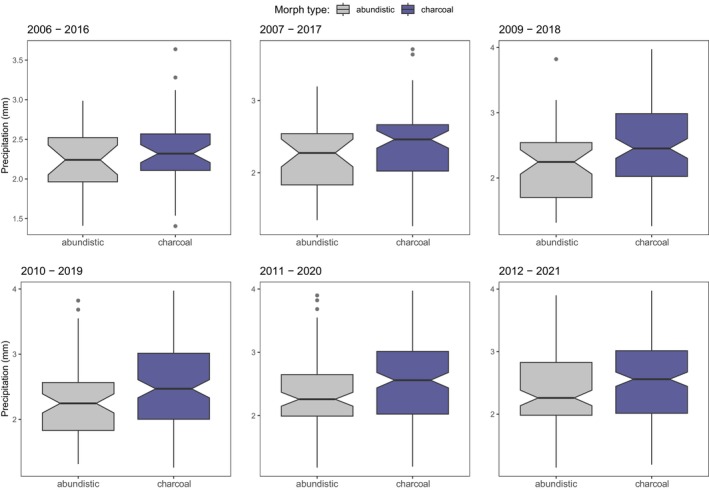
Morph type selection regulated by precipitation patterns in *H. viridiflavus*. Here, we show that the relationship between precipitation patterns and morph types does not change over time, where charcoal is overall favored in high‐precipitation areas, and vice versa for abundistic. Note that no data are available for 2008.

Finally, we evaluated whether these relationships between UV radiation and precipitation patterns are consistent over time. We found that in *N. helvetica* UV radiation consistently favors the charcoal morph (Figure 5), and that this relationship is highly significant across the examined periods (Figure 5; Tables S16–S21). Interestingly, the UV coefficient estimates increase over time (Tables S16–S21) suggesting an amplified influence of UV in selecting morph types in more recent periods. Moreover, while charcoal occurs throughout the UV range, picturata is bounded to lower UV levels, indicating different tradeoffs and selection pressures between morphs. Similarly, in *H. viridiflavus* precipitation patterns consistently favor the charcoal morph (Figure 6), but this relationship is not significantly consistent over time, and only the last two time periods show significant effects (Figure 6; Tables S22–S27).

## 4 DISCUSSION

In this study, we examined the intraspecific color variation within barred grass snakes (*N. helvetica*) and green whip snakes (*H. viridiflavus*) across the Italian peninsula and its relationship with climatic factors on field observation spanning nearly two decades. We specifically focused on melanism variation between the “charcoal” and “picturata” morphs in *N. helvetica* (Bruni et al., 2022), and “charcoal” and “abundistic” morphs (uniform black and abundistic black coloration, respectively) in *H. viridiflavus*. While some of our expectations were not supported—such as picturata and abundistic morphs thriving in humid environments for concealment and charcoal morphs being favored in cold environments for heat absorption—we found significant correlations. Specifically, high UV radiation was linked to the prevalence of charcoal morphs in *N. helvetica*, and high‐precipitation patterns were associated with the prevalence of charcoal morphs in *H. viridiflavus*. Low UV radiation was associated with a higher occurrence of picturata morph and low precipitations with the abundistic morph. Our findings shed light on the correlative effects of climate variables in shaping melanism across different species of snakes, suggesting that multiple factors influence the responses of local populations to changing environments, which ultimately offer valuable insights to understand how microscale variations may contribute to macroevolutionary processes of species.

Microevolutionary processes, characterized by changes in the frequency of a gene in a population due to mutation, selection, gene flow, or genetic drift (Harding et al., 2019), play a fundamental role in elucidating and understanding macroevolutionary patterns and the broader tapestry of biodiversity. As these processes may function at distinct levels of biological organization and operate across different spatial and temporal scales, it is important to consider microevolution in conjunction with macroevolution when discerning the factors that have influenced the evolution of species (Saupe & Myers, 2021). Our investigation into the intraspecific color variation within *N. helvetica* and *H. viridiflavus* across the Italian peninsula provides a tangible example of how microevolutionary processes interact with environmental factors to drive responses in natural populations. Microscale processes provide insights into how populations respond to localized selective pressures, resulting in the emergence of distinct traits and genetic adaptations (Mimura et al., 2017). In our study, we found significant correlations between UV radiation levels and morph selection in *N. helvetica*, with high‐UV radiation favoring the charcoal morph and low UV radiation favoring the picturata morph. By contrast, precipitation patterns significantly influenced morph selection in *H. viridiflavus*, with high precipitation favoring the charcoal morph and low precipitation favoring the abundistic morph. Over time, the accumulation of these microevolutionary changes can give rise to macroevolutionary events, such as speciation, that underpin the diversification of life on Earth. For instance, insects are some of the best‐known examples of how gradual microevolutionary variations can lead to significant changes such as the development of larger mandibles (Moczek & Kijimoto, 2014), pesticide resistance (Hawkins et al., 2019), and the expression of new colors (Hochkirch et al., 2008). Understanding how intraspecific variation in traits, behavior, and genetics interacts with environmental factors is crucial for deciphering the mechanisms driving adaptation at both micro and macro levels because we can deepen the knowledge of the dynamic nature of evolutionary processes.

Similarly, in our study, we also found a strong influence of local climate conditions in shaping the melanistic morphs of snakes. Such microscale variations in coloration, driven by an immediate environmental factor, possibly reflect the adaptive responses of local populations and different life‐history strategies of species. As melanin can act as a protection mechanism against strong UV radiation, we speculate that a plain, black dorsal coloration in *N. helvetica* provides higher protection in such environments. Furthermore, the charcoal morph shares its range with the picturata morph, but the reverse is not true (Figure 1). Although melanin production costs are still unclear (Roulin, 2016), this observation suggests a potential tradeoff, where speckled melanin may be less energetically expensive than uniform dorsal coloration. Interestingly, this relationship remained consistent over time, indicating a persistent influence of UV radiation on morph selection (Figure 5). Our results also showed that the coefficient estimates for UV radiation increased over time, suggesting an amplified influence of UV in selecting morph types in more recent periods, hinting at ongoing, local response processes. Such consistent patterns of trait associations within our study highlight the potential influence of microlevel trait variation on the macroevolution of species. However, melanin not only provides a protection mechanism against harmful UV radiation (Jablonski & Chaplin, 2010) but also against microbial infections (Mackintosh, 2001; Nappi & Christensen, 2005). The charcoal morph in *H. viridiflavus* was associated with high‐precipitation patterns, possibly providing a shield mechanism in very humid environments against external pathogens. Interestingly, this association was not consistent over time and as strong as UV radiation in *N. helvetica*. This suggests a time lag in ecological responses, where the response of the charcoal morph to precipitation changes might operate with a delay. It is possible that the full impact of increased precipitation on the charcoal morph's distribution is not immediately apparent and may manifest gradually over an extended period. Alternatively, the charcoal morph might have initially responded to specific precipitation conditions, but environmental changes over time could be leading to shifts in its responses. For example, it might be adjusting to a broader range of precipitation conditions. Furthermore, if the charcoal morph in *H. viridiflavus* is controlled by relatively few genes, genetic drift might cause fluctuations in its frequency within the population, leading to inconsistent patterns over time. Although Storniolo et al. (2023) have recently suggested that founder effects might exert a stronger selection on diverse color patterns in *H. viridiflavus* than climate variables, their study primarily focused on temperature without considering other climatic factors. As our findings incorporate multiple climate variables and demonstrate a significant effect of precipitation on morph type selection, further examinations are warranted to corroborate the underlying selection pressures of this species.

While UV radiation and precipitation patterns emerged as a significant drivers of morph selection, other environmental factors, such as temperature, wind speed, and elevation, did not exhibit significant influence on the prevalence of charcoal, picturata, or abundistic morphs. This suggests that, in the case of the *N. helvetica* and *H. viridiflavus*, UV radiation, and precipitation patterns likely serve as a particularly potent selective force, potentially overshadowing the effects of other climatic variables. This may be linked to the behavioral ecology of these species, where the preference for aquatic habitats in *N. helvetica* (Brown, 1991) and for dry rural and urban areas in *H. viridiflavus* (Fornasiero et al., 2016) may mitigate the influence of climate‐ and environmental‐related factors, except for UV radiation and precipitation, respectively. The absence of a significant influence of the analyzed environmental variables, but UV and precipitation, on morph types underscores the intricate nature of evolutionary responses, wherein different traits may be favored or selected against in response to specific ecological pressures. Nevertheless, in *N. helvetica* our model also showed a significant effect of the intercept indicating that other nonexamined variables shape the morph type. As our analysis focused solely on climate variables, it is worth considering that other abiotic factors and biological and ecological traits, such as activity pattern (Goldenberg et al., 2021), body size (Goldenberg et al., 2023), behavior (Culumber, 2016), species interaction (Kettlewell, 1961), or environmental pollution (melanin can also sequester heavy metals via chelation, offering mitigation mechanisms against polluted habitats; Di Mauro et al., 2017; Dubey & Roulin, 2014), could have additional impacts on the outcome of our models. Exploring these potential interactions between type of melanisms and ecological traits remains an intriguing avenue for future research. Finally, quantifying melanin is important for unraveling the intricate mechanisms by which organisms adapt to their environments (Forsman et al., 2008). Notably, the amount of melanin present on the external body surface is correlated with its internal distribution (Dubey & Roulin, 2014). As such, the physiological roles of internal melanin are more pronounced in dark‐colored individuals than in their light‐colored counterparts, suggesting a potential interdependence between internal melanin and color evolution. While our study primarily focused on the external, observable color features, a more in‐depth exploration of the vertical structuring of melanin could provide fascinating insights into the adaptive significance of this pigment in response to environmental stressors.

## 5 CONCLUSIONS

Overall, our study highlights the importance of considering intraspecific variation when investigating the ecological and evolutionary dynamics of species in response to climate change. It also supports that macroevolutionary patterns, driven by microevolutionary processes, are species‐specific and describing phenotypic variations at the macroscale level requires a thorough examination of local, microenvironmental conditions (Goldenberg et al., 2022). However, it is crucial to note that our findings represent associations and do not imply direct causation. Further research incorporating the quantification of melanin differences and considering unmeasured ecological factors is essential to validate any adaptive conclusions drawn from these associations. Nonetheless, the interplay between environmental factors and intraspecific variation can provide insights into how organisms respond to their local environments. As we navigate an era of rapidly changing climate, understanding these responses becomes critical for the conservation and management of biodiversity. By elucidating the relationship behind microscale color variations in response to climate in *N. helvetica* and *H. viridiflavus*, our research contributes to a deeper understanding of the processes that drive macrolevel variations in these species. Such level of understanding contributes to the growing body of knowledge on the role of intraspecific variation in shaping the evolutionary trajectories of species, ultimately advancing our knowledge of the mechanisms driving their resilience and success in a changing world.

## AUTHOR CONTRIBUTIONS

**J. Goldenberg:** Conceptualization (equal); data curation (equal); formal analysis (lead); funding acquisition (equal); methodology (lead); visualization (lead); writing – original draft (lead); writing – review and editing (lead). **K. Bisschop:** Formal analysis (supporting); funding acquisition (equal); investigation (supporting); methodology (supporting); visualization (supporting); writing – review and editing (supporting). **G. Bruni:** Conceptualization (equal); data curation (equal); resources (supporting); writing – review and editing (supporting). **M. R. Di Nicola:** Conceptualization (supporting); data curation (equal); resources (supporting); writing – review and editing (supporting). **F. Banfi:** Conceptualization (supporting); data curation (equal); investigation (supporting); resources (supporting); writing – review and editing (supporting). **F. P. Faraone:** Conceptualization (supporting); data curation (equal); resources (supporting); writing – review and editing (supporting).

## FUNDING INFORMATION

JG was funded by the Wenner‐Gren Foundations (UPD2022‐0061). KB and FB were funded by the Research Foundation—Flanders (FWO), KB via the junior postdoctoral research grant (12T5622N) and FB via the doctoral (PhD) grant for fundamental research (11I5223N).

## CONFLICT OF INTEREST STATEMENT

The authors declare no conflicts of interest.

## Supporting information


**Data S1:** Supporting Information

## Data Availability

Supporting figures and tables are available in our supplementary materials. Data deposited at https://doi.org/10.5281/zenodo.11663431.
