# Supplementary material for: Melanin‐based color variation in response to changing climates in snakes
Source: Ecol Evol. 2024 Jun 28;14(7):e11627. doi: 10.1002/ece3.11627 (PMC11213819; doi:10.1002/ece3.11627)
Supplement: Supplementary file 1 — Data S1: Supporting Information [file ECE3-14-e11627-s001.docx]

## SUPPLEMENTARY MATERIALS

**to Melanin-based color variation in response to changing climates in snakes**

**by Jonathan Goldenberg, Karen Bisschop, Giacomo Bruni, Matteo Riccardo Di Nicola, Federico Banfi, Francesco Paolo Faraone**

**Supporting figures**


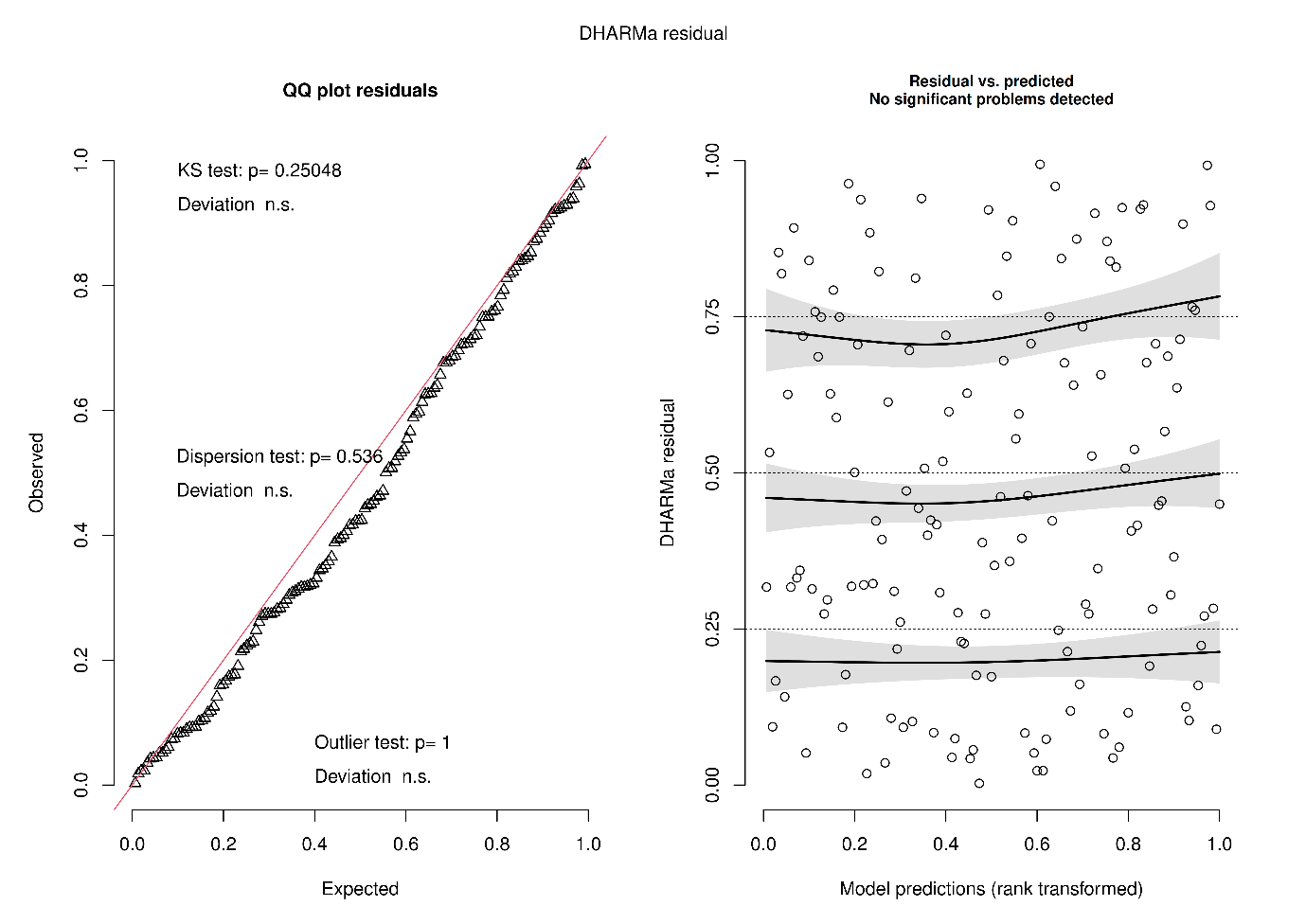


**Figure S1. Model evaluation in *N. helvetica*.** The residual diagnostics for our mixed regression model show that there is no overall deviation from the expected distribution (qq-plot on left panel), and no deviations from the model expectation (plot of the residuals against the predicted value on the right panel).


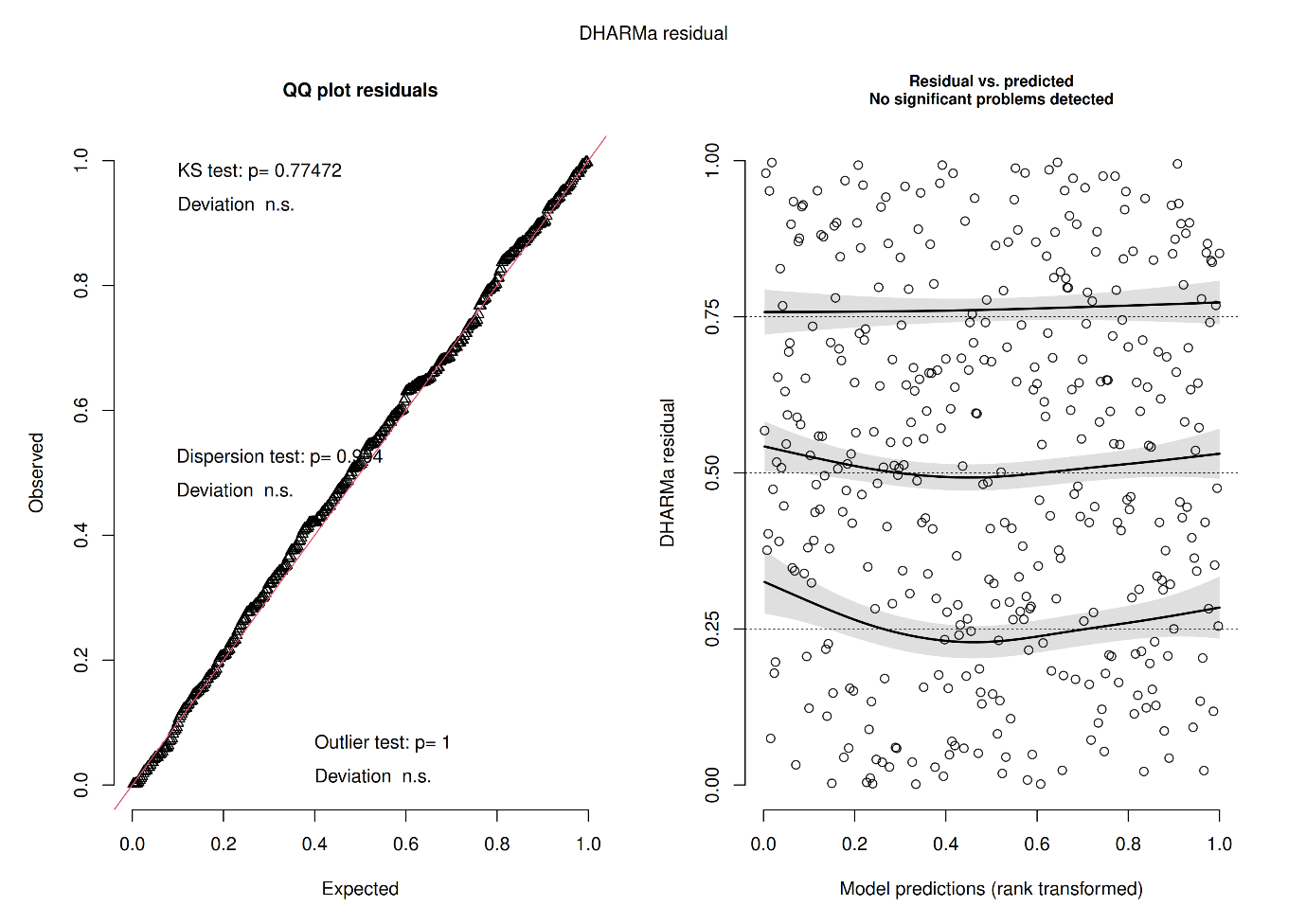


**Figure S2. Model evaluation in *H. viridiflavus*.** The residual diagnostics for our mixed regression model show that there is no overall deviation from the expected distribution (qq-plot on left panel), and no deviations from the model expectation (plot of the residuals against the predicted value on the right panel).


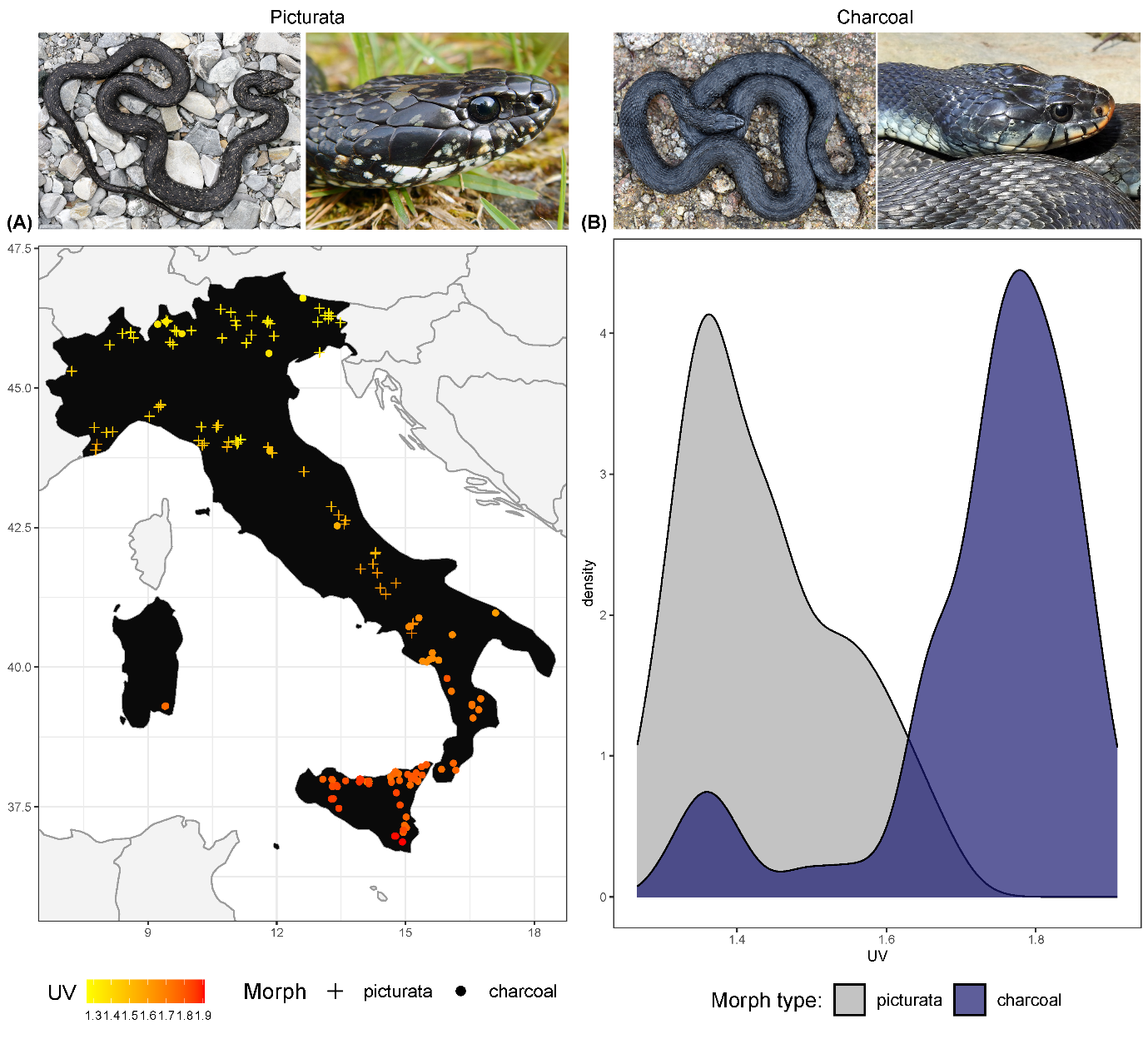


**Figure S3.** Relationship between UV radiation and melanin-rich morph types in *N. helvetica*. **(A)** Across the Italian peninsula, charcoal morphs cluster in the southern regions with stronger UV radiations, whereas picturata with lower UV radiations. **(B)** The density plot shows that charcoal morphs overlap with picturata, and not vice versa, but picturata is selected in lower UV radiation regions. Specimen photos adapted with permission from Bruni et al. (2022).


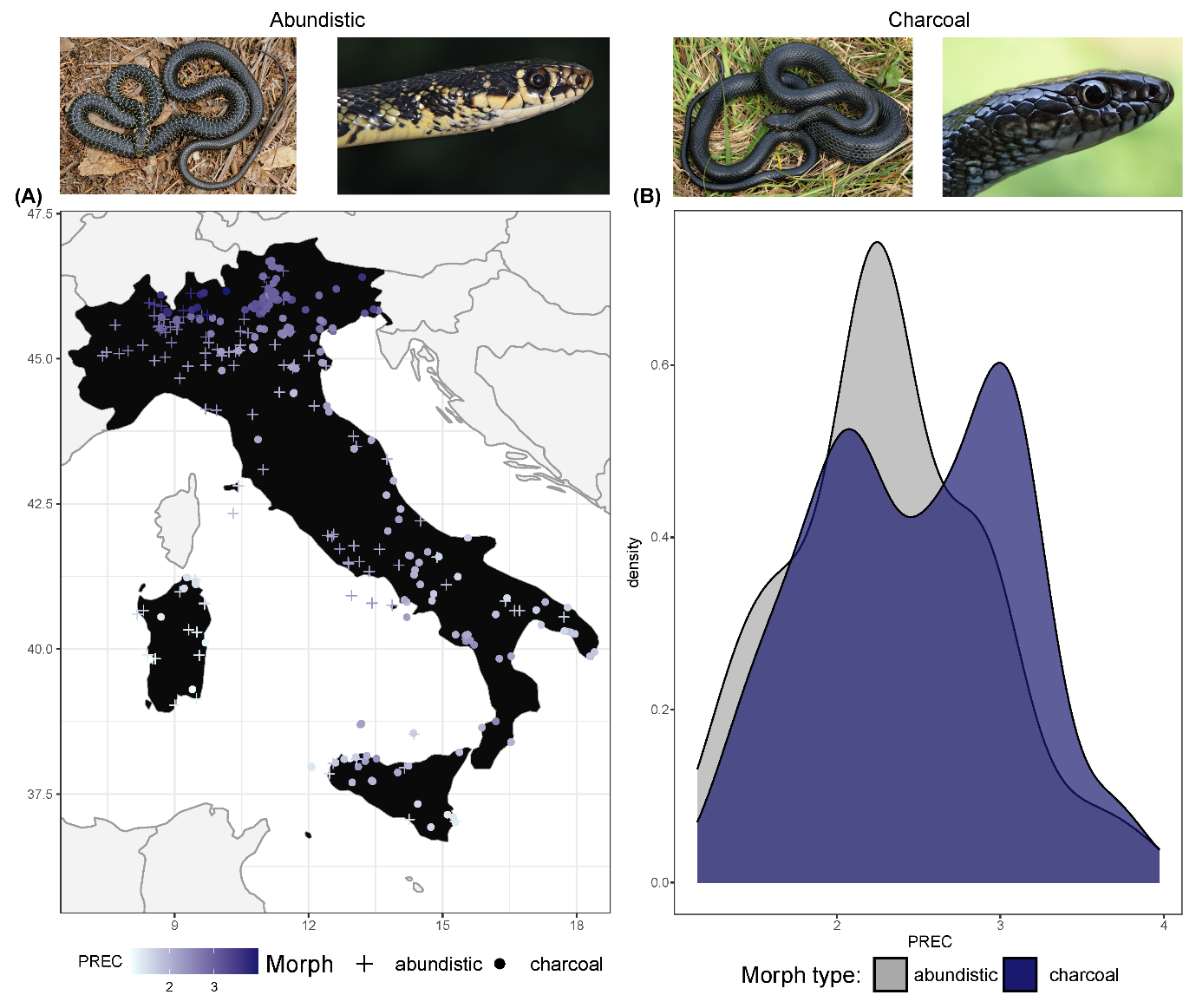


**Figure S4.** Relationship between precipitation patterns and melanin-rich morph types in *H. viridiflavus*. **(A)** Across the Italian peninsula, charcoal morphs cluster in the southern regions and north-eastern ward, whereas abundistic in central islands and north-western ward. **(B)** The density plot shows that charcoal morphs overlap with abundistic, and to a lesser extent vice versa, but abundistic is favored in lower precipitation pattern areas. Specimen photos from MRDN.

**Supporting tables**

**Table S1. Summary of the *Natrix helvetica* spatial model.** Only intercept and UV radiation significantly affect morph type. Temperature measured in °C, UV adimensional, wind speed in m/s, precipitation in mm and elevation in m. The selected family model distribution is binomial. Morph types: picturata (0; speckled black pattern) and charcoal (1; uniform dark coloration). 1.nu: smoothness (ν) parameter, controls the smoothness of the spatial/temporal process.; 1.rho: range (ρ) parameter, controls the distance/range over which points are correlated.

| **Fixed effects** |  |  |  |
| --- | --- | --- | --- |
|  | Estimate | Cond.SE | t-value |
| (Intercept) | -47.6612 | 18.61 | -2.562 |
| Temperature | 0.253455 | 0.19 | 1.344 |
| UV | 22.66097 | 9.27 | 2.445 |
| Wind Speed | 1.587403 | 1.27 | 1.251 |
| Precipitation | 2.092181 | 1.41 | 1.487 |
| Elevation | 0.001218 | 0.00 | 1.361 |

| **Random effects** |  |
| --- | --- |
| Parameter | Value |
| ***Correlation parameters*** |  |
| 1.nu | 16.666667 |
| 1.rho | 4.817947 |
| ***Variance parameters ('lambda')*** |  |
| Longitude + Latitude | 4.323 |
| Year | 0.8997 |

| **χ^2^ test for each term** |  |  |  |  |
| --- | --- | --- | --- | --- |
|  | Df | Chisq. | Pr(>Chisq.) |  |
| (Intercept) | 1 | 6.5622 | 0.01 | * |
| Temperature | 1 | 1.8076 | 0.179 |  |
| UV | 1 | 5.9775 | 0.014 | * |
| Wind Speed | 1 | 1.5652 | 0.211 |  |
| Precipitation | 1 | 2.2112 | 0.137 |  |
| Elevation | 1 | 1.8528 | 0.173 |  |

-----

Significant codes: 0 ‘***’ 0.001 ‘**’ 0.01 ‘*’ 0.05 ‘.’ 0.1 ‘ ’ 1

**Table S2. Summary of the *Hierophis viridiflavus* spatial model.** Only the intercept and precipitation radiation significantly affect morph type. Temperature measured in °C, UV adimensional, wind speed in m/s, precipitation in mm and elevation in m. The selected family model distribution is binomial. Morph types: charcoal (0; uniform dark coloration) and abundistic (1; widening in the dark elements of the dorsal pattern). 1.nu: smoothness (ν) parameter, controls the smoothness of the spatial/temporal process.; 1.rho: range (ρ) parameter, controls the distance/range over which points are correlated.

| **Fixed effects** |  |  |  |
| --- | --- | --- | --- |
|  | Estimate | Cond.SE | t-value |
| (Intercept) | 7.527518 | 5.288021 | 1.4235 |
| Temperature | -0.09351 | 0.103125 | -0.9067 |
| UV | -3.20435 | 4.38131 | -0.7314 |
| Wind Speed | 0.098254 | 0.3717 | 0.2643 |
| Precipitation | -1.47934 | 0.637786 | -2.3195 |
| Elevation | -0.00082 | 0.000663 | -1.2331 |

| **Random effects** |  |
| --- | --- |
| Parameter | Value |
| ***Correlation parameters*** |  |
| 1.nu | 16.666667 |
| 1.rho | 3.443329 |
| ***Variance parameters ('lambda')*** |  |
| Longitude + Latitude | 2.917 |
| Year | 0.08388 |

| **χ^2^ test for each term** |  |  |  |  |
| --- | --- | --- | --- | --- |
|  | Df | Chisq. | Pr(>Chisq.) |  |
| (Intercept) | 1 | 2.0264 | 0.15459 |  |
| Temperature | 1 | 0.8222 | 0.36455 |  |
| UV | 1 | 0.5349 | 0.46455 |  |
| Wind Speed | 1 | 0.0699 | 0.79152 |  |
| Precipitation | 1 | 5.3801 | 0.02037 | * |
| Elevation | 1 | 1.5206 | 0.21752 |  |

-----

Significant codes: 0 ‘***’ 0.001 ‘**’ 0.01 ‘*’ 0.05 ‘.’ 0.1 ‘ ’ 1

**Table S3. Summary of the *N. helvetica* spatial model using T_min_.** No significant effects are found with this model composition. Temperature measured in °C, UV adimensional, wind speed in m/s, precipitation in mm and elevation in m. The selected family model distribution is binomial. Morph types: picturata (0; speckled black pattern) and charcoal (1; uniform dark coloration). 1.nu: smoothness (ν) parameter, controls the smoothness of the spatial/temporal process.; 1.rho: range (ρ) parameter, controls the distance/range over which points are correlated.

| **Fixed effects** |  |  |  |
| --- | --- | --- | --- |
|  | Estimate | Cond.SE | t-value |
| (Intercept) | 1510.046 | 1245 | 1.2124 |
| Temperature_min | -9.4065 | 23.75 | -0.396 |
| UV | -747.365 | 606.8 | -1.2316 |
| Wind Speed | -29.1425 | 123.4 | -0.2362 |
| Precipitation | -92.1886 | 99.43 | -0.9272 |
| Elevation | -0.0254 | 0.09862 | -0.2575 |

| **Random effects** |  |
| --- | --- |
| Parameter | Value |
| ***Correlation parameters*** |  |
| 1.nu | 1.40683 |
| 1.rho | 235.21703 |
| ***Variance parameters ('lambda')*** |  |
| Longitude + Latitude | 13180 |
| Year | 24.83 |

| **χ^2^ test for each term** |  |  |  |  |
| --- | --- | --- | --- | --- |
|  | Df | Chisq. | Pr(>Chisq.) |  |
| (Intercept) | 1 | 1.47003 | 0.2253 |  |
| Temperature_min | 1 | 0.15685 | 0.6921 |  |
| UV | 1 | 1.51688 | 0.2181 |  |
| Wind Speed | 1 | 0.0558 | 0.8133 |  |
| Precipitation | 1 | 0.85962 | 0.3538 |  |
| Elevation | 1 | 0.0663 | 0.7968 |  |

-----

Significant codes: 0 ‘***’ 0.001 ‘**’ 0.01 ‘*’ 0.05 ‘.’ 0.1 ‘ ’ 1

**Table S4. Summary of the *N. helvetica* spatial model using T_max_.** No significant effects are found with this model composition. Temperature measured in °C, UV adimensional, wind speed in m/s, precipitation in mm and elevation in m. The selected family model distribution is binomial. Morph types: picturata (0; speckled black pattern) and charcoal (1; uniform dark coloration). 1.nu: smoothness (ν) parameter, controls the smoothness of the spatial/temporal process.; 1.rho: range (ρ) parameter, controls the distance/range over which points are correlated.

| **Fixed effects** |  |  |  |
| --- | --- | --- | --- |
|  | Estimate | Cond.SE | t-value |
| (Intercept) | 797.6 | 1740 | 0.4583 |
| Temperature_max | 0.5705 | 28.54 | 0.01999 |
| UV | -448.9 | 663.5 | -0.67649 |
| Wind Speed | -19.51 | 106.9 | -0.18248 |
| Precipitation | -39.03 | 137.5 | -0.28389 |
| Elevation | 0.009885 | 0.08034 | 0.12305 |

| **Random effects** |  |
| --- | --- |
| Parameter | Value |
| ***Correlation parameters*** |  |
| 1.nu | 0.0781537 |
| 1.rho | 38.6174664 |
| ***Variance parameters ('lambda')*** |  |
| Longitude + Latitude | 18280 |
| Year | 63.39 |

| **χ^2^ test for each term** |  |  |  |  |
| --- | --- | --- | --- | --- |
|  | Df | Chisq. | Pr(>Chisq.) |  |
| (Intercept) | 1 | 0.21004 | 0.6467 |  |
| Temperature_max | 1 | 0.0004 | 0.9841 |  |
| UV | 1 | 0.45764 | 0.4987 |  |
| Wind Speed | 1 | 0.0333 | 0.8552 |  |
| Precipitation | 1 | 0.08059 | 0.7765 |  |
| Elevation | 1 | 0.01514 | 0.9021 |  |

-----

Significant codes: 0 ‘***’ 0.001 ‘**’ 0.01 ‘*’ 0.05 ‘.’ 0.1 ‘ ’ 1

**Table S5. Summary of the *N. helvetica* spatial model using the interaction T_min_:Precipitation.** No significant effects are found with this model composition. Temperature measured in °C, UV adimensional, wind speed in m/s, precipitation in mm and elevation in m. The selected family model distribution is binomial. Morph types: picturata (0; speckled black pattern) and charcoal (1; uniform dark coloration). 1.nu: smoothness (ν) parameter, controls the smoothness of the spatial/temporal process.; 1.rho: range (ρ) parameter, controls the distance/range over which points are correlated.

| **Fixed effects** |  |  |  |
| --- | --- | --- | --- |
|  | Estimate | Cond.SE | t-value |
| (Intercept) | 204.3 | 236.1043 | 0.86529 |
| Temperature_min | -4.919 | 6.59909 | -0.74537 |
| Precipitation | -17.37 | 21.78668 | -0.79734 |
| UV | -93.23 | 115.2576 | -0.80885 |
| Wind Speed | 2.316 | 20.66957 | 0.11207 |
| Elevation | -0.00031 | 0.01729 | -0.01806 |
| Temperature_min:Precipitation | 1.253 | 2.73817 | 0.4576 |

| **Random effects** |  |
| --- | --- |
| Parameter | Value |
| ***Correlation parameters*** |  |
| 1.nu | 1.120043 |
| 1.rho | 273.338792 |
| ***Variance parameters ('lambda')*** |  |
| Longitude + Latitude | 752.9 |
| Year | 0.00005252 |

| **χ^2^ test for each term** |  |  |  |  |
| --- | --- | --- | --- | --- |
|  | Df | Chisq. | Pr(>Chisq.) |  |
| (Intercept) | 1 | 0.74873 | 0.3869 |  |
| Temperature_min | 1 | 0.55557 | 0.4561 |  |
| Precipitation | 1 | 0.63575 | 0.4253 |  |
| UV | 1 | 0.65424 | 0.4186 |  |
| Wind Speed | 1 | 0.01256 | 0.9108 |  |
| Elevation | 1 | 0.00033 | 0.9856 |  |
| Temperature_min:Precipitation | 1 | 0.2094 | 0.6472 |  |

-----

Significant codes: 0 ‘***’ 0.001 ‘**’ 0.01 ‘*’ 0.05 ‘.’ 0.1 ‘ ’ 1

**Table S6. Summary of the *N. helvetica* spatial model using the interaction T_max_:Precipitation.** No significant effects are found with this model composition. Temperature measured in °C, UV adimensional, wind speed in m/s, precipitation in mm and elevation in m. The selected family model distribution is binomial. Morph types: picturata (0; speckled black pattern) and charcoal (1; uniform dark coloration). 1.nu: smoothness (ν) parameter, controls the smoothness of the spatial/temporal process.; 1.rho: range (ρ) parameter, controls the distance/range over which points are correlated.

| **Fixed effects** |  |  |  |
| --- | --- | --- | --- |
|  | Estimate | Cond.SE | t-value |
| (Intercept) | 327.8 | 342.7302 | 0.95646 |
| Temperature_max | -1.597 | 13.0545 | -0.12231 |
| Precipitation | -22.32 | 112.9455 | -0.19761 |
| UV | -162.8 | 184.3075 | -0.88324 |
| Wind Speed | -1.994 | 28.41654 | -0.07017 |
| Elevation | 0.005919 | 0.01806 | 0.32771 |
| Temperature_max:Precipitation | 0.3455 | 4.96695 | 0.06956 |

| **Random effects** |  |
| --- | --- |
| Parameter | Value |
| ***Correlation parameters*** |  |
| 1.nu | 0.005000525 |
| 1.rho | 52.951237667 |
| ***Variance parameters ('lambda')*** |  |
| Longitude + Latitude | 883.6 |
| Year | 35.58 |

| **χ^2^ test for each term** |  |  |  |  |
| --- | --- | --- | --- | --- |
|  | Df | Chisq. | Pr(>Chisq.) |  |
| (Intercept) | 1 | 0.91482 | 0.3388 |  |
| Temperature_max | 1 | 0.01496 | 0.9026 |  |
| Precipitation | 1 | 0.03905 | 0.8433 |  |
| UV | 1 | 0.78011 | 0.3771 |  |
| Wind Speed | 1 | 0.00492 | 0.9441 |  |
| Elevation | 1 | 0.10739 | 0.7431 |  |
| Temperature_max:Precipitation | 1 | 0.00484 | 0.9445 |  |

-----

Significant codes: 0 ‘***’ 0.001 ‘**’ 0.01 ‘*’ 0.05 ‘.’ 0.1 ‘ ’ 1

**Table S7. Summary of the *H. viridiflavus* spatial model using T_min_.** Precipitation is the only significant predictor with this model composition. Temperature measured in °C, UV adimensional, wind speed in m/s, precipitation in mm and elevation in m. The selected family model distribution is binomial. Morph types: charcoal (0; uniform dark coloration) and abundistic (1; widening in the dark elements of the dorsal pattern). 1.nu: smoothness (ν) parameter, controls the smoothness of the spatial/temporal process.; 1.rho: range (ρ) parameter, controls the distance/range over which points are correlated.

| **Fixed effects** |  |  |  |
| --- | --- | --- | --- |
|  | Estimate | Cond.SE | t-value |
| (Intercept) | -6.59155 | 5.09919 | -1.2927 |
| Temperature_min | 0.0596 | 0.088947 | 0.6701 |
| UV | 3.440175 | 4.347194 | 0.7914 |
| Wind Speed | -0.12285 | 0.438989 | -0.2798 |
| Precipitation | 1.317355 | 0.588841 | 2.2372 |
| Elevation | 0.00076 | 0.000657 | 1.1569 |

| **Random effects** |  |
| --- | --- |
| Parameter | Value |
| ***Correlation parameters*** |  |
| 1.nu | 16.666667 |
| 1.rho | 3.424655 |
| ***Variance parameters ('lambda')*** |  |
| Longitude + Latitude | 2.85 |
| Year | 0.07974 |

| **χ^2^ test for each term** |  |  |  |  |
| --- | --- | --- | --- | --- |
|  | Df | Chisq. | Pr(>Chisq.) |  |
| (Intercept) | 1 | 1.671 | 0.19613 |  |
| Temperature_min | 1 | 0.449 | 0.50282 |  |
| UV | 1 | 0.6262 | 0.42874 |  |
| Wind Speed | 1 | 0.0783 | 0.77959 |  |
| Precipitation | 1 | 5.0051 | 0.02527 | * |
| Elevation | 1 | 1.3384 | 0.24731 |  |

-----

Significant codes: 0 ‘***’ 0.001 ‘**’ 0.01 ‘*’ 0.05 ‘.’ 0.1 ‘ ’ 1

**Table S8. Summary of the *H. viridiflavus* spatial model using T_max_.** Precipitation is the only significant predictor with this model composition. Temperature measured in °C, UV adimensional, wind speed in m/s, precipitation in mm and elevation in m. The selected family model distribution is binomial. Morph types: charcoal (0; uniform dark coloration) and abundistic (1; widening in the dark elements of the dorsal pattern). 1.nu: smoothness (ν) parameter, controls the smoothness of the spatial/temporal process.; 1.rho: range (ρ) parameter, controls the distance/range over which points are correlated.

| **Fixed effects** |  |  |  |
| --- | --- | --- | --- |
|  | Estimate | Cond.SE | t-value |
| (Intercept) | -10.69 | 6.357248 | -1.6815 |
| Temperature_max | 0.09331 | 0.08036 | 1.1612 |
| UV | 3.553 | 4.339853 | 0.8187 |
| Wind Speed | 0.2572 | 0.352445 | 0.7297 |
| Precipitation | 1.779 | 0.745054 | 2.3874 |
| Elevation | 0.000802 | 0.000643 | 1.2461 |

| **Random effects** |  |
| --- | --- |
| Parameter | Value |
| ***Correlation parameters*** |  |
| 1.nu | 16.666667 |
| 1.rho | 3.446543 |
| ***Variance parameters ('lambda')*** |  |
| Longitude + Latitude | 2.832 |
| Year | 0.0779 |

| **χ^2^ test for each term** |  |  |  |  |
| --- | --- | --- | --- | --- |
|  | Df | Chisq. | Pr(>Chisq.) |  |
| (Intercept) | 1 | 2.8275 | 0.09266 | . |
| Temperature_max | 1 | 1.3483 | 0.24557 |  |
| UV | 1 | 0.6702 | 0.41299 |  |
| Wind Speed | 1 | 0.5324 | 0.4656 |  |
| Precipitation | 1 | 5.6999 | 0.01697 | * |
| Elevation | 1 | 1.5528 | 0.21273 |  |

-----

Significant codes: 0 ‘***’ 0.001 ‘**’ 0.01 ‘*’ 0.05 ‘.’ 0.1 ‘ ’ 1

**Table S9. Summary of the *H. viridiflavus* spatial model using the interaction T_min_:Precipitation.** No significant effects are found with this model composition. Temperature measured in °C, UV adimensional, wind speed in m/s, precipitation in mm and elevation in m. The selected family model distribution is binomial. Morph types: charcoal (0; uniform dark coloration) and abundistic (1; widening in the dark elements of the dorsal pattern). 1.nu: smoothness (ν) parameter, controls the smoothness of the spatial/temporal process.; 1.rho: range (ρ) parameter, controls the distance/range over which points are correlated.

| **Fixed effects** |  |  |  |
| --- | --- | --- | --- |
|  | Estimate | Cond.SE | t-value |
| (Intercept) | -5.73023 | 5.260125 | -1.0894 |
| Temperature_min | -0.27131 | 0.301652 | -0.8994 |
| Precipitation | 0.539395 | 0.897735 | 0.6008 |
| UV | 4.594275 | 4.553449 | 1.009 |
| Wind Speed | 0.224208 | 0.545707 | 0.4109 |
| Elevation | 0.00065 | 0.000667 | 0.9745 |
| Temperature_min:Precipitation | 0.114048 | 0.098431 | 1.1587 |

| **Random effects** |  |
| --- | --- |
| Parameter | Value |
| ***Correlation parameters*** |  |
| 1.nu | 16.666667 |
| 1.rho | 3.445106 |
| ***Variance parameters ('lambda')*** |  |
| Longitude + Latitude | 3.049 |
| Year | 0.07955 |

| **χ^2^ test for each term** |  |  |  |  |
| --- | --- | --- | --- | --- |
|  | Df | Chisq. | Pr(>Chisq.) |  |
| (Intercept) | 1 | 1.18673 | 0.276 |  |
| Temperature_min | 1 | 0.80895 | 0.3684 |  |
| Precipitation | 1 | 0.36101 | 0.5479 |  |
| UV | 1 | 1.01801 | 0.313 |  |
| Wind Speed | 1 | 0.1688 | 0.6812 |  |
| Elevation | 1 | 0.94972 | 0.3298 |  |
| Temperature_min:Precipitation | 1 | 1.3425 | 0.2466 |  |

-----

Significant codes: 0 ‘***’ 0.001 ‘**’ 0.01 ‘*’ 0.05 ‘.’ 0.1 ‘ ’ 1

**Table S10. Summary of the *H. viridiflavus* spatial model using the interaction T_max_:Precipitation.** No significant effects are found with this model composition. Temperature measured in °C, UV adimensional, wind speed in m/s, precipitation in mm and elevation in m. The selected family model distribution is binomial. Morph types: charcoal (0; uniform dark coloration) and abundistic (1; widening in the dark elements of the dorsal pattern). 1.nu: smoothness (ν) parameter, controls the smoothness of the spatial/temporal process.; 1.rho: range (ρ) parameter, controls the distance/range over which points are correlated.

| **Fixed effects** |  |  |  |
| --- | --- | --- | --- |
|  | Estimate | Cond.SE | t-value |
| (Intercept) | -4.70099 | 8.099677 | -0.5804 |
| Temperature_max | -0.1627 | 0.216375 | -0.7519 |
| Precipitation | -1.03369 | 2.320287 | -0.4455 |
| UV | 4.580506 | 4.53892 | 1.0092 |
| Wind Speed | 0.173766 | 0.368245 | 0.4719 |
| Elevation | 0.000899 | 0.000656 | 1.3704 |
| Temperature_max:Precipitation | 0.108242 | 0.084147 | 1.2863 |

| **Random effects** |  |
| --- | --- |
| Parameter | Value |
| ***Correlation parameters*** |  |
| 1.nu | 16.666667 |
| 1.rho | 3.494718 |
| ***Variance parameters ('lambda')*** |  |
| Longitude + Latitude | 3.126 |
| Year | 0.07626 |

| **χ^2^ test for each term** |  |  |  |  |
| --- | --- | --- | --- | --- |
|  | Df | Chisq. | Pr(>Chisq.) |  |
| (Intercept) | 1 | 0.33686 | 0.5616 |  |
| Temperature_max | 1 | 0.56539 | 0.4521 |  |
| Precipitation | 1 | 0.19847 | 0.656 |  |
| UV | 1 | 1.01841 | 0.3129 |  |
| Wind Speed | 1 | 0.22267 | 0.637 |  |
| Elevation | 1 | 1.87805 | 0.1706 |  |
| Temperature_max:Precipitation | 1 | 1.65469 | 0.1983 |  |

-----

Significant codes: 0 ‘***’ 0.001 ‘**’ 0.01 ‘*’ 0.05 ‘.’ 0.1 ‘ ’ 1

**Table S11. Summary of the *N. helvetica* spatial model using humidity.** No significant effects are found with this model composition. Temperature measured in °C, UV adimensional, wind speed in m/s, humidity as % and elevation in m. The selected family model distribution is binomial. Morph types: picturata (0; speckled black pattern) and charcoal (1; uniform dark coloration). 1.nu: smoothness (ν) parameter, controls the smoothness of the spatial/temporal process.; 1.rho: range (ρ) parameter, controls the distance/range over which points are correlated.

| **Fixed effects** |  |  |  |
| --- | --- | --- | --- |
|  | Estimate | Cond.SE | t-value |
| (Intercept) | 339.1 | 213.3226 | 1.5898 |
| Temperature_min | -0.9302 | 3.02271 | -0.30773 |
| UV | -109.7 | 66.47596 | -1.65006 |
| Wind Speed | -6.248 | 12.81003 | -0.48777 |
| Humidity | -1.992 | 1.82097 | -1.094 |
| Elevation | -0.00076 | 0.01102 | -0.06922 |

| **Random effects** |  |
| --- | --- |
| Parameter | Value |
| ***Correlation parameters*** |  |
| 1.nu | 1.40683 |
| 1.rho | 235.21703 |
| ***Variance parameters ('lambda')*** |  |
| Longitude + Latitude | 0.07674397 |
| Year | 1.29511459 |

| **χ^2^ test for each term** |  |  |  |  |
| --- | --- | --- | --- | --- |
|  | Df | Chisq. | Pr(>Chisq.) |  |
| (Intercept) | 1 | 2.52747 | 0.11188 |  |
| Temperature_min | 1 | 0.0947 | 0.75829 |  |
| UV | 1 | 2.7227 | 0.09893 | . |
| Wind Speed | 1 | 0.23792 | 0.62571 |  |
| Humidity | 1 | 1.19683 | 0.27396 |  |
| Elevation | 1 | 0.00479 | 0.94481 |  |

-----

Significant codes: 0 ‘***’ 0.001 ‘**’ 0.01 ‘*’ 0.05 ‘.’ 0.1 ‘ ’ 1

**Table S12. Summary of the *N. helvetica* spatial model using the interaction temperature: humidity.** No significant effects are found with this model composition. Temperature measured in °C, UV adimensional, wind speed in m/s, humidity as % and elevation in m. The selected family model distribution is binomial. Morph types: picturata (0; speckled black pattern) and charcoal (1; uniform dark coloration). 1.nu: smoothness (ν) parameter, controls the smoothness of the spatial/temporal process.; 1.rho: range (ρ) parameter, controls the distance/range over which points are correlated.

| **Fixed effects** |  |  |  |
| --- | --- | --- | --- |
|  | Estimate | Cond.SE | t-value |
| (Intercept) | 280.4 | 442.3 | 0.63393 |
| Temperature_min | -1.929 | 21.57 | -0.08941 |
| Humidity | -1.649 | 5.654 | -0.2917 |
| UV | -92.56 | 59.9 | -1.54539 |
| Wind Speed | -5.61 | 12.09 | -0.46394 |
| Elevation | 0.000555 | 0.009878 | 0.05618 |
| Temperature_min: Humidity | 0.02009 | 0.2873 | 0.06993 |

| **Random effects** |  |
| --- | --- |
| Parameter | Value |
| ***Correlation parameters*** |  |
| 1.nu | 0.07228381 |
| 1.rho | 1.42415046 |
| ***Variance parameters ('lambda')*** |  |
| Longitude + Latitude | 336.4 |
| Year | 72.33 |

| **χ^2^ test for each term** |  |  |  |  |
| --- | --- | --- | --- | --- |
|  | Df | Chisq. | Pr(>Chisq.) |  |
| (Intercept) | 1 | 0.40186 | 0.5261 |  |
| Temperature_min | 1 | 0.00799 | 0.9288 |  |
| Humidity | 1 | 0.08509 | 0.7705 |  |
| UV | 1 | 2.38822 | 0.1223 |  |
| Wind Speed | 1 | 0.21524 | 0.6427 |  |
| Elevation | 1 | 0.00316 | 0.9552 |  |
| Temperature_min: Humidity | 1 | 0.00489 | 0.9443 |  |

-----

Significant codes: 0 ‘***’ 0.001 ‘**’ 0.01 ‘*’ 0.05 ‘.’ 0.1 ‘ ’ 1

**Table S13. Summary of the *H. viridiflavus* spatial model using humidity.** No significant effects are found with this model composition. Temperature measured in °C, UV adimensional, wind speed in m/s, humidity as % and elevation in m. The selected family model distribution is binomial. Morph types: charcoal (0; uniform dark coloration) and abundistic (1; widening in the dark elements of the dorsal pattern). 1.nu: smoothness (ν) parameter, controls the smoothness of the spatial/temporal process.; 1.rho: range (ρ) parameter, controls the distance/range over which points are correlated.

| **Fixed effects** |  |  |  |
| --- | --- | --- | --- |
|  | Estimate | Cond.SE | t-value |
| (Intercept) | -3.42301 | 9.078745 | -0.377 |
| Temperature_min | 0.025595 | 0.126747 | 0.2019 |
| UV | 0.716617 | 4.322061 | 0.1658 |
| Wind Speed | -0.08751 | 0.427038 | -0.2049 |
| Humidity | 0.036333 | 0.100621 | 0.3611 |
| Elevation | 0.000915 | 0.000641 | 1.4279 |

| **Random effects** |  |
| --- | --- |
| Parameter | Value |
| ***Correlation parameters*** |  |
| 1.nu | 16.666667 |
| 1.rho | 3.362265 |
| ***Variance parameters ('lambda')*** |  |
| Longitude + Latitude | 3.054 |
| Year | 0.08167 |

| **χ^2^ test for each term** |  |  |  |  |
| --- | --- | --- | --- | --- |
|  | Df | Chisq. | Pr(>Chisq.) |  |
| (Intercept) | 1 | 0.14216 | 0.7061 |  |
| Temperature_min | 1 | 0.04078 | 0.84 |  |
| UV | 1 | 0.02749 | 0.8683 |  |
| Wind Speed | 1 | 0.042 | 0.8376 |  |
| Humidity | 1 | 0.13038 | 0.718 |  |
| Elevation | 1 | 2.03899 | 0.1533 |  |

-----

Significant codes: 0 ‘***’ 0.001 ‘**’ 0.01 ‘*’ 0.05 ‘.’ 0.1 ‘ ’ 1

**Table S14. Summary of the *H. viridiflavus* spatial model using the interaction temperature: humidity.** Temperature and its interaction with humidity are marginally influencing morph types. Temperature measured in °C, UV adimensional, wind speed in m/s, humidity as % and elevation in m. The selected family model distribution is binomial. Morph types: charcoal (0; uniform dark coloration) and abundistic (1; widening in the dark elements of the dorsal pattern). 1.nu: smoothness (ν) parameter, controls the smoothness of the spatial/temporal process.; 1.rho: range (ρ) parameter, controls the distance/range over which points are correlated.

| **Fixed effects** |  |  |  |
| --- | --- | --- | --- |
|  | Estimate | Cond.SE | t-value |
| (Intercept) | 33.54543 | 20.15 | 1.6646 |
| Temperature_min | -2.51791 | 1.269 | -1.9845 |
| Humidity | -0.43478 | 0.2567 | -1.6936 |
| UV | 1.029759 | 5.048 | 0.204 |
| Wind Speed | 0.062881 | 0.4297 | 0.1463 |
| Elevation | 0.00066 | 0.000642 | 1.0279 |
| Temperature_min: Humidity | 0.032428 | 0.01621 | 2.0003 |

| **Random effects** |  |
| --- | --- |
| Parameter | Value |
| ***Correlation parameters*** |  |
| 1.nu | 2.0195099 |
| 1.rho | 0.6325295 |
| ***Variance parameters ('lambda')*** |  |
| Longitude + Latitude | 5.01 |
| Year | 0.06052 |

| **χ^2^ test for each term** |  |  |  |  |
| --- | --- | --- | --- | --- |
|  | Df | Chisq. | Pr(>Chisq.) |  |
| (Intercept) | 1 | 2.771 | 0.09598 | . |
| Temperature_min | 1 | 3.9383 | 0.0472 | * |
| Humidity | 1 | 2.8682 | 0.09035 | . |
| UV | 1 | 0.0416 | 0.83837 |  |
| Wind Speed | 1 | 0.0214 | 0.88365 |  |
| Elevation | 1 | 1.0565 | 0.30401 |  |
| Temperature_min: Humidity | 1 | 4.0013 | 0.04547 | * |

-----

Significant codes: 0 ‘***’ 0.001 ‘**’ 0.01 ‘*’ 0.05 ‘.’ 0.1 ‘ ’ 1

**Table S.15 Summary of the *N. helvetica* and *H. viridiflavus* spatial models accounting for the interaction term.** As the models show diverging results, the absence of temperature-precipitation interaction in our study does not explain the disparities observed between species.

*N. helvetica H. viridiflavus*

| **Fixed effects** |  |  |  |  | **Fixed effects** | Estimate | Cond. SE | t-value |
| --- | --- | --- | --- | --- | --- | --- | --- | --- |
|  | Estimate | Cond. SE | t-value |  |  |  |  |  |
| (Intercept) | -47.5103 | 18.56 | -2.5594 |  | (Intercept) | 4.020221 | 6.282913 | 0.6399 |
| Temperature | 0.220555 | 0.32 | 0.6893 |  | Temperature | 0.333265 | 0.394785 | 0.8442 |
| Precipitation | 1.815543 | 2.44 | 0.7448 |  | Precipitation | 0.248042 | 1.663645 | 0.1491 |
| UV | 22.88455 | 9.34 | 2.4502 |  | UV | -5.0061 | 4.7822 | -1.0468 |
| Wind Speed | 1.547774 | 1.27 | 1.2204 |  | Wind Speed | -0.17158 | 0.453191 | -0.3786 |
| Elevation | 0.001256 | 0.00 | 1.3294 |  | Elevation | -0.00067 | 0.000674 | -0.9999 |
| Temperature:  Precipitation | 0.021261 | 0.15 | 0.1441 |  | Temperature:  Precipitation | -0.13822 | 0.122098 | -1.132 |

| **χ^2^ test** | |  |  |  |  | **χ^2^ test** |  |  |  |
| --- | --- | --- | --- | --- | --- | --- | --- | --- | --- |
|  | Df | Chisq. | Pr(>Chisq.) | |  |  | Df | Chisq. | Pr(>Chisq.) |
| (Intercept) | 1 | 6.5506 | 0.01048 | * |  | (Intercept) | 1 | 0.40943 | 0.5223 |
| Temperature | 1 | 0.4751 | 0.49065 |  |  | Temperature | 1 | 0.71262 | 0.3986 |
| Precipitation | 1 | 0.5547 | 0.45642 |  |  | Precipitation | 1 | 0.02223 | 0.8815 |
| UV | 1 | 6.0035 | 0.01428 | * |  | UV | 1 | 1.09583 | 0.2952 |
| Wind Speed | 1 | 1.4894 | 0.2223 |  |  | Wind Speed | 1 | 0.14334 | 0.705 |
| Elevation | 1 | 1.7674 | 0.18371 |  |  | Elevation | 1 | 0.99971 | 0.3174 |
| Temperature:  Precipitation | 1 | 0.0208 | 0.88538 |  |  | Temperature:  Precipitation | 1 | 1.28147 | 0.2576 |

**Table S16. Summary of the *N. helvetica* spatial model for the temporal window 2007-2016.** Only UV significantly affects morph type in this time period. Temperature measured in °C, UV adimensional, wind speed in m/s, humidity as % and elevation in m. The selected family model distribution is binomial. Morph types: picturata (0; speckled black pattern) and charcoal (1; uniform dark coloration). 1.nu: smoothness (ν) parameter, controls the smoothness of the spatial/temporal process.; 1.rho: range (ρ) parameter, controls the distance/range over which points are correlated.

| **Fixed effects** |  |  |  |
| --- | --- | --- | --- |
|  | Estimate | Cond.SE | t-value |
| (Intercept) | 218.8111 | 175.146 | 1.2493 |
| Temperature | -2.50517 | 4.06619 | -0.6161 |
| UV | -14.34 | 3.939 | -3.640 |
| Wind Speed | -1.72196 | 13.18554 | -0.1306 |
| Precipitation | -12.7191 | 11.28302 | -1.1273 |
| Elevation | -0.01056 | 0.01066 | -0.9907 |

| **Random effects** |  |
| --- | --- |
| Parameter | Value |
| ***Correlation parameters*** |  |
| 1.nu | 0.0050 |
| 1.rho | 107.5437 |
| ***Variance parameters ('lambda')*** |  |
| Longitude + Latitude | 139.1 |
| Year | 0.4287 |

| **χ^2^ test for each term** |  |  |  |  |
| --- | --- | --- | --- | --- |
|  | Df | Chisq. | Pr(>Chisq.) |  |
| (Intercept) | 1 | 1.56077 | 0.2116 |  |
| Temperature | 1 | 0.37958 | 0.5378 |  |
| UV | 1 | 13.251 | 0.0002724 | ** |
| Wind Speed | 1 | 0.01705 | 0.8961 |  |
| Precipitation | 1 | 1.27076 | 0.2596 |  |
| Elevation | 1 | 0.98145 | 0.3218 |  |

-----

Significant codes: 0 ‘***’ 0.001 ‘**’ 0.01 ‘*’ 0.05 ‘.’ 0.1 ‘ ’ 1

**Table S17. Summary of the *N. helvetica* spatial model for the temporal window 2008-2017.** Only UV significantly affects morph type in this time period. Temperature measured in °C, UV adimensional, wind speed in m/s, humidity as % and elevation in m. The selected family model distribution is binomial. Morph types: picturata (0; speckled black pattern) and charcoal (1; uniform dark coloration). 1.nu: smoothness (ν) parameter, controls the smoothness of the spatial/temporal process.; 1.rho: range (ρ) parameter, controls the distance/range over which points are correlated.

| **Fixed effects** |  |  |  |
| --- | --- | --- | --- |
|  | Estimate | Cond.SE | t-value |
| (Intercept) | 401.2527 | 346.36533 | 1.1585 |
| Temperature | -7.0322 | 7.77792 | -0.9041 |
| UV | -15.56 | 4.216 | -3.689 |
| Wind Speed | 13.6794 | 25.44255 | 0.5377 |
| Precipitation | -22.2043 | 22.48746 | -0.9874 |
| Elevation | -0.0173 | 0.02189 | -0.79 |

| **Random effects** |  |
| --- | --- |
| Parameter | Value |
| ***Correlation parameters*** |  |
| 1.nu | 0.2824827 |
| 1.rho | 136.0058772 |
| ***Variance parameters ('lambda')*** |  |
| Longitude + Latitude | 509.9 |
| Year | 0.00323 |

| **χ^2^ test for each term** |  |  |  |  |
| --- | --- | --- | --- | --- |
|  | Df | Chisq. | Pr(>Chisq.) |  |
| (Intercept) | 1 | 1.34204 | 0.2467 |  |
| Temperature | 1 | 0.81745 | 0.3659 |  |
| UV | 1 | 13.611 | 0.0002249 | *** |
| Wind Speed | 1 | 0.28908 | 0.5908 |  |
| Precipitation | 1 | 0.97497 | 0.3234 |  |
| Elevation | 1 | 0.62417 | 0.4295 |  |

-----

Significant codes: 0 ‘***’ 0.001 ‘**’ 0.01 ‘*’ 0.05 ‘.’ 0.1 ‘ ’ 1

**Table S18. Summary of the *N. helvetica* spatial model for the temporal window 2009-2018.** Only UV significantly affects morph type in this time period. Temperature measured in °C, UV adimensional, wind speed in m/s, humidity as % and elevation in m. The selected family model distribution is binomial. Morph types: picturata (0; speckled black pattern) and charcoal (1; uniform dark coloration). 1.nu: smoothness (ν) parameter, controls the smoothness of the spatial/temporal process.; 1.rho: range (ρ) parameter, controls the distance/range over which points are correlated.

| **Fixed effects** |  |  |  |
| --- | --- | --- | --- |
|  | Estimate | Cond.SE | t-value |
| (Intercept) | 262.6 | 233.45163 | 1.125 |
| Temperature | -0.5157 | 3.1616 | -0.1631 |
| UV | -18.46 | 5.285 | -3.492 |
| Wind Speed | 4.268 | 26.30212 | 0.1623 |
| Precipitation | -11.19 | 16.51292 | -0.6775 |
| Elevation | -0.005083 | 0.01382 | -0.3677 |

| **Random effects** |  |
| --- | --- |
| Parameter | Value |
| ***Correlation parameters*** |  |
| 1.nu | 0.00607427 |
| 1.rho | 120.00893143 |
| ***Variance parameters ('lambda')*** |  |
| Longitude + Latitude | 487.7 |
| Year | 18.75 |

| **χ^2^ test for each term** |  |  |  |  |
| --- | --- | --- | --- | --- |
|  | Df | Chisq. | Pr(>Chisq.) |  |
| (Intercept) | 1 | 1.26569 | 0.2606 |  |
| Temperature | 1 | 0.02661 | 0.8704 |  |
| UV | 1 | 12.197 | 0.0004788 | *** |
| Wind Speed | 1 | 0.02633 | 0.8711 |  |
| Precipitation | 1 | 0.45904 | 0.4981 |  |
| Elevation | 1 | 0.13522 | 0.7131 |  |

-----

Significant codes: 0 ‘***’ 0.001 ‘**’ 0.01 ‘*’ 0.05 ‘.’ 0.1 ‘ ’ 1

**Table S19. Summary of the *N. helvetica* spatial model for the temporal window 2010-2019.** Only UV, precipitation and the intercept significantly affect morph type in this time period. Temperature measured in °C, UV adimensional, wind speed in m/s, humidity as % and elevation in m. The selected family model distribution is binomial. Morph types: picturata (0; speckled black pattern) and charcoal (1; uniform dark coloration). 1.nu: smoothness (ν) parameter, controls the smoothness of the spatial/temporal process.; 1.rho: range (ρ) parameter, controls the distance/range over which points are correlated.

| **Fixed effects** |  |  |  |
| --- | --- | --- | --- |
|  | Estimate | Cond.SE | t-value |
| (Intercept) | 56.4 | 18.01 | 3.131 |
| Temperature | -0.2084 | 0.1393 | -1.496 |
| UV | -20.6 | 5.802 | -3.458 |
| Wind Speed | -0.3168 | 1.568 | -0.202 |
| Precipitation | -2.588 | 1.256 | -2.06 |
| Elevation | -0.000894 | 0.000879 | -1.017 |

| **Random effects** |  |
| --- | --- |
| Parameter | Value |
| ***Correlation parameters*** |  |
| 1.nu | 16.666667 |
| 1.rho | 6.206711 |
| ***Variance parameters ('lambda')*** |  |
| Longitude + Latitude | 5.2 |
| Year | 1.154 |

| **χ^2^ test for each term** |  |  |  |  |
| --- | --- | --- | --- | --- |
|  | Df | Chisq. | Pr(>Chisq.) |  |
| (Intercept) | 1 | 9.8041 | 0.001741 | ** |
| Temperature | 1 | 2.2387 | 0.13459 |  |
| UV | 1 | 8.5917 | 0.003377 | ** |
| Wind Speed | 1 | 0.0408 | 0.839938 |  |
| Precipitation | 1 | 4.244 | 0.03939 | * |
| Elevation | 1 | 1.0337 | 0.309286 |  |

-----

Significant codes: 0 ‘***’ 0.001 ‘**’ 0.01 ‘*’ 0.05 ‘.’ 0.1 ‘ ’ 1

**Table S20. Summary of the *N. helvetica* spatial model for the temporal window 2011-2020.** Only UV significantly affects morph type in this time period. Temperature measured in °C, UV adimensional, wind speed in m/s, humidity as % and elevation in m. The selected family model distribution is binomial. Morph types: picturata (0; speckled black pattern) and charcoal (1; uniform dark coloration). 1.nu: smoothness (ν) parameter, controls the smoothness of the spatial/temporal process.; 1.rho: range (ρ) parameter, controls the distance/range over which points are correlated.

| **Fixed effects** |  |  |  |
| --- | --- | --- | --- |
|  | Estimate | Cond.SE | t-value |
| (Intercept) | 410.4 | 460.2705 | 0.89164 |
| Temperature | -0.7384 | 8.11336 | -0.09101 |
| UV | -20.89 | 5.983 | -3.492 |
| Wind Speed | 5.775 | 65.06984 | 0.08875 |
| Precipitation | -18.76 | 33.20094 | -0.56496 |
| Elevation | 0.000889 | 0.02694 | 0.033 |

| **Random effects** |  |
| --- | --- |
| Parameter | Value |
| ***Correlation parameters*** |  |
| 1.nu | 0.5758346 |
| 1.rho | 5.772693 |
| ***Variance parameters ('lambda')*** |  |
| Longitude + Latitude | 733 |
| Year | 38.31 |

| **χ^2^ test for each term** |  |  |  |  |
| --- | --- | --- | --- | --- |
|  | Df | Chisq. | Pr(>Chisq.) |  |
| (Intercept) | 1 | 0.79503 | 0.3726 |  |
| Temperature | 1 | 0.00828 | 0.9275 |  |
| UV | 1 | 12.193 | 0.0004797 | *** |
| Wind Speed | 1 | 0.00788 | 0.9293 |  |
| Precipitation | 1 | 0.31918 | 0.5721 |  |
| Elevation | 1 | 0.00109 | 0.9737 |  |

-----

Significant codes: 0 ‘***’ 0.001 ‘**’ 0.01 ‘*’ 0.05 ‘.’ 0.1 ‘ ’ 1

**Table S21. Summary of the *N. helvetica* spatial model for the temporal window 2012-2021.** Only UV significantly affects morph type in this time period. Temperature measured in °C, UV adimensional, wind speed in m/s, humidity as % and elevation in m. The selected family model distribution is binomial. Morph types: picturata (0; speckled black pattern) and charcoal (1; uniform dark coloration). 1.nu: smoothness (ν) parameter, controls the smoothness of the spatial/temporal process.; 1.rho: range (ρ) parameter, controls the distance/range over which points are correlated.

| **Fixed effects** |  |  |  |
| --- | --- | --- | --- |
|  | Estimate | Cond.SE | t-value |
| (Intercept) | 284 | 239.21686 | 1.18734 |
| Temperature | -0.4751 | 4.6888 | -0.10132 |
| UV | -21.22 | 5.921 | -3.584 |
| Wind Speed | 4.659 | 30.03475 | 0.15514 |
| Precipitation | -12.29 | 16.52832 | -0.74357 |
| Elevation | -0.000275 | 0.01454 | -0.01891 |

| **Random effects** |  |
| --- | --- |
| Parameter | Value |
| ***Correlation parameters*** |  |
| 1.nu | 0.0069442 |
| 1.rho | 5.894758 |
| ***Variance parameters ('lambda')*** |  |
| Longitude + Latitude | 5.201 |
| Year | 2.255 |

| **χ^2^ test for each term** |  |  |  |  |
| --- | --- | --- | --- | --- |
|  | Df | Chisq. | Pr(>Chisq.) |  |
| (Intercept) | 1 | 1.40978 | 0.2351 |  |
| Temperature | 1 | 0.01027 | 0.9193 |  |
| UV | 1 | 12.846 | 0.0003382 | *** |
| Wind Speed | 1 | 0.02407 | 0.8767 |  |
| Precipitation | 1 | 0.55289 | 0.4571 |  |
| Elevation | 1 | 0.00036 | 0.9849 |  |

-----

Significant codes: 0 ‘***’ 0.001 ‘**’ 0.01 ‘*’ 0.05 ‘.’ 0.1 ‘ ’ 1

**Table S22. Summary of the *H. viridiflavus* spatial model for the temporal window 2006-2016.** No significant effects are found for this time period. Temperature measured in °C, UV adimensional, wind speed in m/s, precipitation in mm and elevation in m. The selected family model distribution is binomial. Morph types: charcoal (0; uniform dark coloration) and abundistic (1; widening in the dark elements of the dorsal pattern). 1.nu: smoothness (ν) parameter, controls the smoothness of the spatial/temporal process.; 1.rho: range (ρ) parameter, controls the distance/range over which points are correlated.

| **Fixed effects** |  |  |  |
| --- | --- | --- | --- |
|  | Estimate | Cond.SE | t-value |
| (Intercept) | 2.872751 | 12.14797 | 0.2365 |
| Temperature | -0.07966 | 0.310751 | -0.2563 |
| UV | -4.24482 | 11.13281 | -0.3813 |
| Wind Speed | 0.192952 | 0.972238 | 0.1985 |
| Precipitation | 1.338306 | 1.425987 | 0.9385 |
| Elevation | 0.001082 | 0.002303 | 0.4701 |

| **Random effects** |  |
| --- | --- |
| Parameter | Value |
| ***Correlation parameters*** |  |
| 1.nu | 4.5325580 |
| 1.rho | 0.8408264 |
| ***Variance parameters ('lambda')*** |  |
| Longitude + Latitude | 15.05 |
| Year | 0.000001 |

| **χ^2^ test for each term** |  |  |  |  |
| --- | --- | --- | --- | --- |
|  | Df | Chisq. | Pr(>Chisq.) |  |
| (Intercept) | 1 | 0.05592 | 0.8131 |  |
| Temperature | 1 | 0.06571 | 0.7977 |  |
| UV | 1 | 0.14538 | 0.703 |  |
| Wind Speed | 1 | 0.03939 | 0.8427 |  |
| Precipitation | 1 | 0.8808 | 0.348 |  |
| Elevation | 1 | 0.22095 | 0.6383 |  |

-----

Significant codes: 0 ‘***’ 0.001 ‘**’ 0.01 ‘*’ 0.05 ‘.’ 0.1 ‘ ’ 1

**Table S23. Summary of the *H. viridiflavus* spatial model for the temporal window 2007-2017.** No significant effects are found for this time period. Temperature measured in °C, UV adimensional, wind speed in m/s, precipitation in mm and elevation in m. The selected family model distribution is binomial. Morph types: charcoal (0; uniform dark coloration) and abundistic (1; widening in the dark elements of the dorsal pattern). 1.nu: smoothness (ν) parameter, controls the smoothness of the spatial/temporal process.; 1.rho: range (ρ) parameter, controls the distance/range over which points are correlated.

| **Fixed effects** |  |  |  |
| --- | --- | --- | --- |
|  | Estimate | Cond.SE | t-value |
| (Intercept) | -1.437126 | 8.362598 | -0.17185 |
| Temperature | 0.049711 | 0.207113 | 0.24002 |
| UV | -1.517754 | 7.153456 | -0.21217 |
| Wind Speed | -0.039535 | 0.712666 | -0.05548 |
| Precipitation | 1.205897 | 1.056122 | 1.14182 |
| Elevation | 0.001506 | 0.001582 | 0.95226 |

| **Random effects** |  |
| --- | --- |
| Parameter | Value |
| ***Correlation parameters*** |  |
| 1.nu | 16.666667 |
| 1.rho | 3.233308 |
| ***Variance parameters ('lambda')*** |  |
| Longitude + Latitude | 4.745 |
| Year | 0. 000001 |

| **χ^2^ test for each term** |  |  |  |  |
| --- | --- | --- | --- | --- |
|  | Df | Chisq. | Pr(>Chisq.) |  |
| (Intercept) | 1 | 0.02953 | 0.8636 |  |
| Temperature | 1 | 0.05761 | 0.8103 |  |
| UV | 1 | 0.04502 | 0.832 |  |
| Wind Speed | 1 | 0.00308 | 0.9558 |  |
| Precipitation | 1 | 1.30374 | 0.2535 |  |
| Elevation | 1 | 0.90679 | 0.341 |  |

-----

Significant codes: 0 ‘***’ 0.001 ‘**’ 0.01 ‘*’ 0.05 ‘.’ 0.1 ‘ ’ 1

**Table S24. Summary of the *H. viridiflavus* spatial model for the temporal window 2009-2018.** No significant effects are found for this time period. Temperature measured in °C, UV adimensional, wind speed in m/s, precipitation in mm and elevation in m. The selected family model distribution is binomial. Morph types: charcoal (0; uniform dark coloration) and abundistic (1; widening in the dark elements of the dorsal pattern). 1.nu: smoothness (ν) parameter, controls the smoothness of the spatial/temporal process.; 1.rho: range (ρ) parameter, controls the distance/range over which points are correlated.

| **Fixed effects** |  |  |  |
| --- | --- | --- | --- |
|  | Estimate | Cond.SE | t-value |
| (Intercept) | -1.237149 | 7.396673 | -0.1673 |
| Temperature | -0.138658 | 0.173352 | -0.7999 |
| UV | 0.6692747 | 5.96499 | 0.1122 |
| Wind Speed | 0.1815353 | 0.572518 | 0.3171 |
| Precipitation | 1.240178 | 0.952454 | 1.3021 |
| Elevation | 0.0003832 | 0.001081 | 0.3544 |

| **Random effects** |  |
| --- | --- |
| Parameter | Value |
| ***Correlation parameters*** |  |
| 1.nu | 16.66667 |
| 1.rho | 3.39011 |
| ***Variance parameters ('lambda')*** |  |
| Longitude + Latitude | 3.395 |
| Year | 0. 000001 |

| **χ^2^ test for each term** |  |  |  |  |
| --- | --- | --- | --- | --- |
|  | Df | Chisq. | Pr(>Chisq.) |  |
| (Intercept) | 1 | 0.02798 | 0.8672 |  |
| Temperature | 1 | 0.63979 | 0.4238 |  |
| UV | 1 | 0.01259 | 0.9107 |  |
| Wind Speed | 1 | 0.10054 | 0.7512 |  |
| Precipitation | 1 | 1.69543 | 0.1929 |  |
| Elevation | 1 | 0.12558 | 0.7231 |  |

-----

Significant codes: 0 ‘***’ 0.001 ‘**’ 0.01 ‘*’ 0.05 ‘.’ 0.1 ‘ ’ 1

**Table S25. Summary of the *H. viridiflavus* spatial model for the temporal window 2010-2019.** No significant effects are found for this time period. Temperature measured in °C, UV adimensional, wind speed in m/s, precipitation in mm and elevation in m. The selected family model distribution is binomial. Morph types: charcoal (0; uniform dark coloration) and abundistic (1; widening in the dark elements of the dorsal pattern). 1.nu: smoothness (ν) parameter, controls the smoothness of the spatial/temporal process.; 1.rho: range (ρ) parameter, controls the distance/range over which points are correlated.

| **Fixed effects** |  |  |  |
| --- | --- | --- | --- |
|  | Estimate | Cond.SE | t-value |
| (Intercept) | -0.44778 | 7.303303 | -0.06131 |
| Temperature | -0.03154 | 0.139839 | -0.22552 |
| UV | -0.35933 | 6.022863 | -0.05966 |
| Wind Speed | 0.072411 | 0.517502 | 0.13992 |
| Precipitation | 0.825821 | 0.916479 | 0.90108 |
| Elevation | 0.000331 | 0.000918 | 0.36096 |

| **Random effects** |  |
| --- | --- |
| Parameter | Value |
| ***Correlation parameters*** |  |
| 1.nu | 16.666667 |
| 1.rho | 3.413708 |
| ***Variance parameters ('lambda')*** |  |
| Longitude + Latitude | 4.67 |
| Year | 0.004471 |

| **χ^2^ test for each term** |  |  |  |  |
| --- | --- | --- | --- | --- |
|  | Df | Chisq. | Pr(>Chisq.) |  |
| (Intercept) | 1 | 0.00376 | 0.9511 |  |
| Temperature | 1 | 0.05086 | 0.8216 |  |
| UV | 1 | 0.00356 | 0.9524 |  |
| Wind Speed | 1 | 0.01958 | 0.8887 |  |
| Precipitation | 1 | 0.81195 | 0.3675 |  |
| Elevation | 1 | 0.13029 | 0.7181 |  |

-----

Significant codes: 0 ‘***’ 0.001 ‘**’ 0.01 ‘*’ 0.05 ‘.’ 0.1 ‘ ’ 1

**Table S26. Summary of the *H. viridiflavus* spatial model for the temporal window 2011-2020.** Precipitation is the only significant predictor within this time period. Temperature measured in °C, UV adimensional, wind speed in m/s, precipitation in mm and elevation in m. The selected family model distribution is binomial. Morph types: charcoal (0; uniform dark coloration) and abundistic (1; widening in the dark elements of the dorsal pattern). 1.nu: smoothness (ν) parameter, controls the smoothness of the spatial/temporal process.; 1.rho: range (ρ) parameter, controls the distance/range over which points are correlated.

| **Fixed effects** |  |  |  |
| --- | --- | --- | --- |
|  | Estimate | Cond.SE | t-value |
| (Intercept) | -5.5921 | 5.949681 | -0.9399 |
| Temperature | 0.1478 | 0.111482 | 1.3258 |
| UV | 1.272081 | 4.924496 | 0.2583 |
| Wind Speed | -0.25048 | 0.423599 | -0.5913 |
| Precipitation | 1.434345 | 0.724647 | 1.9794 |
| Elevation | 0.00071 | 0.000723 | 0.9808 |

| **Random effects** |  |
| --- | --- |
| Parameter | Value |
| ***Correlation parameters*** |  |
| 1.nu | 16.666667 |
| 1.rho | 3.394699 |
| ***Variance parameters ('lambda')*** |  |
| Longitude + Latitude | 3.258 |
| Year | 0.0657 |

| **χ^2^ test for each term** |  |  |  |  |
| --- | --- | --- | --- | --- |
|  | Df | Chisq. | Pr(>Chisq.) |  |
| (Intercept) | 1 | 0.8834 | 0.34727 |  |
| Temperature | 1 | 1.7577 | 0.18491 |  |
| UV | 1 | 0.0667 | 0.79616 |  |
| Wind Speed | 1 | 0.3497 | 0.55431 |  |
| Precipitation | 1 | 3.9179 | 0.04777 | * |
| Elevation | 1 | 0.962 | 0.32668 |  |

-----

Significant codes: 0 ‘***’ 0.001 ‘**’ 0.01 ‘*’ 0.05 ‘.’ 0.1 ‘ ’ 1

**Table S27. Summary of the *H. viridiflavus* spatial model for the temporal window 2012-2021.** Precipitation is the only significant predictor within this time period. Temperature measured in °C, UV adimensional, wind speed in m/s, precipitation in mm and elevation in m. The selected family model distribution is binomial. Morph types: charcoal (0; uniform dark coloration) and abundistic (1; widening in the dark elements of the dorsal pattern). 1.nu: smoothness (ν) parameter, controls the smoothness of the spatial/temporal process.; 1.rho: range (ρ) parameter, controls the distance/range over which points are correlated.

| **Fixed effects** |  |  |  |
| --- | --- | --- | --- |
|  | Estimate | Cond.SE | t-value |
| (Intercept) | -7.677887 | 5.4335442 | -1.41305 |
| Temperature | 0.083844 | 0.1029048 | 0.81477 |
| UV | 3.331575 | 4.4881643 | 0.7423 |
| Wind Speed | -0.007211 | 0.3757384 | -0.01919 |
| Precipitation | 1.452746 | 0.6450584 | 2.25212 |
| Elevation | 0.000757 | 0.0006657 | 1.13719 |

| **Random effects** |  |
| --- | --- |
| Parameter | Value |
| ***Correlation parameters*** |  |
| 1.nu | 16.666667 |
| 1.rho | 3.441654 |
| ***Variance parameters ('lambda')*** |  |
| Longitude + Latitude | 2.824 |
| Year | 0.07979 |

| **χ^2^ test for each term** |  |  |  |  |
| --- | --- | --- | --- | --- |
|  | Df | Chisq. | Pr(>Chisq.) |  |
| (Intercept) | 1 | 1.9967 | 0.15764 |  |
| Temperature | 1 | 0.6639 | 0.4152 |  |
| UV | 1 | 0.551 | 0.4579 |  |
| Wind Speed | 1 | 0.0004 | 0.98469 |  |
| Precipitation | 1 | 5.072 | 0.02432 | * |
| Elevation | 1 | 1.2932 | 0.25546 |  |

-----

Significant codes: 0 ‘***’ 0.001 ‘**’ 0.01 ‘*’ 0.05 ‘.’ 0.1 ‘ ’ 1
